# Supplementary material for: Meniscal Tear Outcome (METRO) review: a protocol for a systematic review summarising the clinical course and patient experiences of meniscal tears in the current literature
Source: BMJ Open. 2020 Aug 4;10(8):e036247. doi: 10.1136/bmjopen-2019-036247 (PMC7406020; doi:10.1136/bmjopen-2019-036247)
Supplement: Supplementary data [file bmjopen-2019-036247supp001.pdf]

Supplementary file

Appendix 1

Search strategy for quantitative aspect of review

Database: Ovid MEDLINE(R) ALL <1946 to September 17, 2019>

Search Strategy:

----

1 exp Tibial Meniscus Injuries/ or exp Meniscus/ (8082)

2 (menisc* adj3 (tear* or injur* or damage*)).mp. [mp=title, abstract, original title, name of substance word, subject heading word, floating sub-heading word, keyword heading word, organism supplementary concept word, protocol supplementary concept word, rare disease supplementary concept word, unique identifier, synonyms] (7547)

3 1 or 2 (10876)

4 arthroscopy.mp. or exp Arthroscopy/ (28087)

5 exp Meniscectomy/ or meniscect*.mp. (3054)

6 (menisc* adj3 repair).mp. [mp=title, abstract, original title, name of substance word, subject heading word, floating sub-heading word, keyword heading word, organism supplementary concept word, protocol supplementary concept word, rare disease supplementary concept word, unique identifier, synonyms] (1565)

7 4 or 5 or 6 (30599)

8 (random$ or placebo$ or single blind$ or double blind$ or triple blind$).mp. [mp=title, abstract, original title, name of substance word, subject heading word, floating sub-heading word, keyword heading word, organism supplementary concept word, protocol supplementary concept word, rare disease supplementary concept word, unique identifier, synonyms] (1389493)

9 exp Randomized Controlled Trial/ (490118)

10 Controlled Clinical Trial/ (93257)

11 exp Clinical Trials as Topic/ (330202)

12 Comparative Study/ (1839704)

13 exp Cohort Studies/ (1898633)

14 Observational Study/ (67005)

15 Prospective Studies/ (513619)

16 8 or 9 or 10 or 11 or 12 or 13 or 14 or 15 (4611873)

17 3 and 7 and 16 (2235)

****

Appendix 2

Search strategy for qualitative aspect of review

Database: Ovid MEDLINE(R) ALL <1946 to September 17, 2019>

Search Strategy:

----

1 exp Tibial Meniscus Injuries/ or exp Meniscus/ (8082)

2 (menisc* adj3 (tear* or injur* or damage*)).mp. [mp=title, abstract, original title, name of substance word, subject heading word, floating sub-heading word, keyword heading word, organism supplementary concept word, protocol supplementary concept word, rare disease supplementary concept word, unique identifier, synonyms] (7547)

3 1 or 2 (10876)

4 health knowledge, attitudes, practice/ or "patient acceptance of health care"/ or patient satisfaction/ (216398)

5 exp Interview/ (28684)

6 exp "Surveys and Questionnaires"/ (975116)

7 exp Focus Groups/ (27525)

8 4 or 5 or 6 or 7 (1157450)

9 (patient$ adj3 (view$ or opinion$ or awareness or tolerance or perception or persistenc$ or attitude$ or compliance or satisfaction or concern$ or belief$ or feeling$ or position or idea$ or preference$ or choice$)).mp. [mp=title, abstract, original title, name of substance word, subject heading word, floating sub-heading word, keyword heading word, organism supplementary concept word, protocol supplementary concept word, rare disease supplementary concept word, unique identifier, synonyms] (268702)

10 (Discomfort or comfort or inconvenience or bother$4 or trouble or fear$ or anxiety or anxious or worr$3).tw. (334544)

11 8 or 9 or 10 (1567999)

12 3 and 11 (723)

****
