# Supplementary material for: Meniscal Tear Outcome (METRO) review: a protocol for a systematic review summarising the clinical course and patient experiences of meniscal tears in the current literature
Source: BMJ Open. 2020 Aug 4;10(8):e036247. doi: 10.1136/bmjopen-2019-036247 (PMC7406020; doi:10.1136/bmjopen-2019-036247)
Supplement: Author's manuscript [file bmjopen-2019-036247.draft_revisions.pdf]

BMJ Open is committed to open peer review. As part of this commitment we make the peer review history of every article we publish publicly available.

When an article is published we post the peer reviewers' comments and the authors' responses online. We also post the versions of the paper that were used during peer review. These are the versions that the peer review comments apply to.

The versions of the paper that follow are the versions that were submitted during the peer review process. They are not the versions of record or the final published versions. They should not be cited or distributed as the published version of this manuscript.

BMJ Open is an open access journal and the full, final, typeset and author-corrected version of record of the manuscript is available on our site with no access controls, subscription charges or pay-per-view fees (http://bmjopen.bmj.com).

If you have any questions on BMJ Open's open peer review process please email info.bmjopen@bmj.com

BMJ Open

# The Meniscal tear outcome (METRO) review: A protocol for a systematic review summarising the natural history and patient experiences of meniscal tears in the current literature.

| | |
|---|---|
| Journal: | *BMJ Open* |
| Manuscript ID | bmjopen-2019-036247 |
| Article Type: | Protocol |
| Date Submitted by the Author: | 06-Dec-2019 |
| Complete List of Authors: | Ahmed, Imran; University of Warwick, Clinical Trials Unit<br>Khatri, Chetan<br>Parsons, Nicholas; University of Warwick, Warwick Medical School<br>Hutchinson, Charles; University of Warwick, Warwick Medical School<br>Staniszewska, Sophie; University of Warwick, Warwick Medical School<br>Price, Andrew; University of Oxford, Nuffield Department of Orthopaedics, Rheumatology and Musculoskeletal Sciences<br>Metcalfe, Andrew; University of Warwick, Warwick Clinical trials Unit |
| Keywords: | Knee < ORTHOPAEDIC & TRAUMA SURGERY, Orthopaedic & trauma surgery < SURGERY, Musculoskeletal disorders < ORTHOPAEDIC & TRAUMA SURGERY |
| | |

SCHOLARONE™
Manuscripts

*I, the Submitting Author has the right to grant and does grant on behalf of all authors of the Work (as defined in the below author licence), an exclusive licence and/or a non-exclusive licence for contributions from authors who are: i) UK Crown employees; ii) where BMJ has agreed a CC-BY licence shall apply, and/or iii) in accordance with the terms applicable for US Federal Government officers or employees acting as part of their official duties; on a worldwide, perpetual, irrevocable, royalty-free basis to BMJ Publishing Group Ltd ("BMJ") its licensees and where the relevant Journal is co-owned by BMJ to the co-owners of the Journal, to publish the Work in this journal and any other BMJ products and to exploit all rights, as set out in our licence.*

*The Submitting Author accepts and understands that any supply made under these terms is made by BMJ to the Submitting Author unless you are acting as an employee on behalf of your employer or a postgraduate student of an affiliated institution which is paying any applicable article publishing charge ("APC") for Open Access articles. Where the Submitting Author wishes to make the Work available on an Open Access basis (and intends to pay the relevant APC), the terms of reuse of such Open Access shall be governed by a Creative Commons licence – details of these licences and which Creative Commons licence will apply to this Work are set out in our licence referred to above.*

*Other than as permitted in any relevant BMJ Author's Self Archiving Policies, I confirm this Work has not been accepted for publication elsewhere, is not being considered for publication elsewhere and does not duplicate material already published. I confirm all authors consent to publication of this Work and authorise the granting of this licence.*

For peer review only

# The Meniscal tear outcome (METRO) review: A protocol for a systematic review summarising the natural history and patient experiences of meniscal tears in the current literature.

Imran Ahmed (Imran.ahmed4@nhs.net)

Chetan Khatri (chetan.khatri@gmail.com)

Nicholas Parsons (Nick.parsons@warwick.ac.uk)

Charles Hutchinson (c.e.hutchinson@warwick.ac.uk)

Sophie Staniszewska (sophie.staniszewska@warwick.ac.uk)

Andrew Price (Andrew.price@ndorms.ox.ac.uk)

Andrew Metcalfe (a.metcalfe@warwick.ac.uk)

Corresponding Author

Imran Ahmed

Trauma and Orthopaedics

Warwick Clinical trials unit

University Hospital Coventry and Warwickshire

Clifford Bridge Road

Coventry

CV2 2DX

Imran.ahmed4@nhs.net

Orchid ID: 0000-0003-2774-9954

For peer review only

## Abstract

### Introduction

Meniscal tears are a common knee injury with an incidence of 60 per 100,000. Management of meniscal tears can include either non-operative measures or operative procedures such as arthroscopic partial meniscectomy (APM). Despite substantial research evaluating the effectiveness of APM in the recent past, little is known about the natural history or the experiences of patients with a meniscal tear.

### Aim

To summarise the short to long term patterns of variability in outcome in patients with a meniscal tear.

To summarise the evidence reporting experiences of patients with a meniscal tear or their views on outcomes used in meniscal tear research.

### Method and analysis

Two search strategies will be developed in order to identify citations from EMBASE, MEDLINE, AMED, CENTRAL, Web of Science and Sociofile. For the quantitative review we will identify studies reporting patient reported outcomes measures (PROMs) in patients after a meniscal tear. The standardised mean change (SMC) will be used to assess the variation in size of response and summarise the overall response to each treatment option. All studies will undergo a quality assessment using either the Cochrane Risk of Bias or the Newcastle-Ottawa tool.

A qualitative systematic review will be used to identify studies reporting views and experiences of patients with a meniscal tear. All studies will be assessed using the Critical Appraisal Skills Programme (CASP) tool and if sufficient data is present a meta-synthesis will be performed to identify first, second and third order constructs.

### Ethics and Dissemination

Given the nature of this study, no formal ethical approval will be sought. Results from the review will be disseminated at national conferences and will be submitted to a peer reviewed journal for publication. Lay summaries will be freely available via the study twitter page.

**Registration:** Prospero ID CRD42019122179

**Word count:** 2520




For peer review only - http://bmjopen.bmj.com/site/about/guidelines.xhtml

For peer review only

**Article summary: Strengths and limitations**

- Comprehensive nature of review including patients undergoing all management options.
- Analysis plan in keeping with previously published systematic reviews
- Risk of publication bias as only English Language studies are to be included
- Patient and public involvement in the dissemination of results

For peer review only



For peer review only - http://bmjopen.bmj.com/site/about/guidelines.xhtml

## Introduction

Meniscal tears are a common knee injury with an incidence of 60-70 per 100,000 and they account for 70,000 hospital admissions per year in the UK[1,2].The menisci fibrocartilaginous structures are situated within the knee[3]. The principal role of the menisci is to distribute compressive load within the knee joint by increasing the contact area and reducing the contact pressure between the tibia and the femur[3-5]. This may play a crucial role in preventing osteoarthritis. Risk factors for meniscal tears include age, gender, body mass index (BMI), presence of degeneration within the knee and level of activity e.g. participation in sport[3]. Meniscal tears can be asymptomatic or present with symptoms such as knee pain, swelling, stiffness or locking of the knee[3,6,7].

The management options for treatment of meniscal tears include watchful waiting (observation), medical therapy (intra-artciular injections, analgesia), physiotherapy and surgery[8-11]. Recent evidence has questioned the effectiveness of surgery when compared to physiotherapy or other non-operative interventions[12-14]. Despite this evidence, the number of arthroscopies has almost doubled over the last 15 years[15]. Many surgeons and researchers believe surgery may be beneficial in a subset of patients and over recent years there has been substantial growth in the number of studies and systematic reviews in the management of meniscal tears[16-18]. These studies are yet to identify a clear superior treatment option in this patient population, or to identify a true subset that may benefit from surgery.

Research into other musculoskeletal conditions has demonstrated that outcomes steadily improve over time regardless of treatment[19,20]. This could be due to the natural history of the condition or simply regression to the mean. The natural history of a condition is a crucial factor to consider when interpretation of outcomes from interventions of patients with meniscal tears. Prospective studies with well-defined entry criteria and follow up points provide an excellent source of information on the natural history of a condition[19]. Researchers and clinicians need to better understand the natural history of meniscal tears to plan treatment decisions and future research.

In addition to understanding the natural history of meniscal tears, we need to understand patient views and experiences of managing meniscal tears as a form of patient based evidence which we utilise alongside clinical and economic forms of evidence.  Recent treatment guidelines were developed, based on the consensus of 25 international experts, by the British Association for Surgery of the Knee (BASK)[11]. However, no patients were involved in the development of these guidelines. In order to treat meniscal tears more effectively and plan informative research we need to understand patient views and expectations in the treatment of meniscal tears.



For peer review only - http://bmjopen.bmj.com/site/about/guidelines.xhtml

**Objectives**

**Quantitative review**

The objective of the quantitative review is to summarise the short, medium and long term temporal patterns of change in patient reported outcome measures (PROMs) in patients with a meniscal tear.

**Qualitative review**

The objective of the qualitative review is to identify and summarise the evidence on patient experiences of meniscal tears.

For peer review only

**Methods and analysis**

This study protocol was developed in accordance with the PRISMA-P (Preferred Reporting Items for Systematic review and Meta-analysis Protocols) checklist. In addition to this manuscript the protocol has been registered on the PROSPERO database and can be found at https://www.crd.york.ac.uk/PROSPERO/display_record.php?RecordID=122179

**Eligibility criteria**

Quantitative review

Inclusion criteria

- Study design
  - Full text Randomised controlled trials in patients treated for a meniscal tear (for example, comparing surgery or a non-operative intervention versus a comparator group).
  - Prospective cohort studies adjusted for case-mix reporting outcomes in patients being treated for a meniscal tear.
- Studies reporting an established knee related PROMs score for at least three months.
- English language studies only.

Exclusion criteria

- Studies not reporting any of the clinical outcomes selected for this review.



For peer review only - http://bmjopen.bmj.com/site/about/guidelines.xhtml

- Studies reporting outcomes in patients with additional major knee ligament injury or fractures around the knee.
- Abstract or conference publications.

Qualitative review

Inclusion criteria

- Qualitative studies reporting the views and experiences of patients with a meniscal tear, implementing all types of qualitative methodology.
- English language studies only.

Exclusion criteria

- Abstract or conference publications

**Search Strategy and Quality assessment**

Following consultation with a librarian a search strategy was devised (see appendix). The authors aim to search the following databases MEDLINE; Excerpta Medica Database (EMBASE); Allied and Complementary Medicine (AMED); and Cochrane Central Register of Controlled Trials (CENTRAL) using OVID Sp. We will also search Web of science and sociofile for qualitative studies. We will also search reference lists of included studies to identify further citations. All citations will be imported into Endnote X9 reference management software[21] (Clarivate Analytics, Philadelphia, USA). After removal of duplicates, titles and abstracts will be screened according to the inclusion criteria. The full texts of studies potentially meeting the inclusion criteria will be screened by two authors (IA and CK). These authors will independently assess each study and any discrepancies will be addressed by discussion with a senior author (NP or AM).

RCTs will be qualitatively assessed using the Cochrane risk of bias tool[22]. Observational studies will be assessed using the Newcastle-Ottawa scale[23]. Two authors (IA and CK) will independently assess the quality of each included study with discrepancies being resolved following discussion with a senior author (NP or AM).

Each qualitative study will be critically appraised using the Critical Appraisal Skills Programme (CASP) for qualitative studies[24]. Each study will be graded as adequate, partially adequate or inadequate. If rated as inadequate the study will be excluded from the meta-synthesis.

For peer review only



For peer review only - http://bmjopen.bmj.com/site/about/guidelines.xhtml

Two authors (IA and AB) independently assessed the quality of evidence. We used the GRADE considerations (study limitations, consistence of effect, imprecision, indirectness and publication bias) to assess the quality of the body of evidence[25]. Decisions to upgrade or downgrade body of evidence have been clearly stated in the discussion.

**Outcomes**

Primary outcome

The primary outcome measure is knee pain or function measured by at least one of the following validated knee questionnaires : (i) Knee injury and osteoarthritis outcome score (KOOS); (ii) Oxford knee score (OKS); (iii) Western Ontario meniscal evaluation tool (WOMET); (iv) Lysholm score and (v) International Knee Documentation Committee score (IKDC).

**Data extraction**

*Extraction plan for quantitative studies*

For each study we will collect number of patients (in each arm if RCT or non-randomised study) and the intervention type, defined as meniscectomy, meniscal repair, placebo surgery or non-operative. In addition, mean age, gender, BMI, method of diagnosis of tear, imaging findings (presence of OA), patient reported symptoms, whether patient and public involvement (PPI) was reported and means and standard deviations (SDs) of the PROMs score at each time point reported will also be collected. We will also extract information on whether any qualitative data were included in the final study report. If a study does not report mean or SD, then where possible estimates will be calculated from other reported statistics (data summaries), e.g. a test statistic or P value, using standard methods as described in the Cochrane handbook[22]. If data are not represented in a simple numeric format (e.g. in a table or in the study text), but are presented graphically only (e.g. a figure or graph), two authors (IA and CK) will extract the data manually. If failing this we are unable to extract the data we will contact the study authors and request the data.

Data analysis

Outcome scores will be plotted against time from study intervention (e.g. non-operative or surgery) to describe change from baseline (at the point of recruitment) to all follow up time points reported in the treatment arms included. This will provide a simple visual representation of the clinical course of the condition. The non-operative arm e.g. physiotherapy or placebo will provide an insight into



For peer review only - http://bmjopen.bmj.com/site/about/guidelines.xhtml

the natural history of the condition, whereas, the operative arms will provide a graphical representation of the change in outcomes over time in this population.

To determine variation in size of response, the change of outcome will be assessed by calculating the bias-corrected standardised mean change (SMC) at 3, 6, 12, 24 and 36 months. The SMC is calculated by subtracting the follow-up mean score for the PROM of interest from the baseline mean score. This is then divided by the pooled SD and multiplied by the bias correction factor based on group size. Follow up or baseline SD will be used if the pooled SD is not reported and 95% confidence intervals (CI) will be calculated using estimates of the variance of the SMC[26].

Studies using a variety of outcomes scales or PROMs can be pooled as the SMC standardises the measurement of change. As in previously reported meta-analyses we will calculate a combined pooled SMC for each time point using a random effects model[19,20]. We will do this for the meniscectomy arm, meniscal repair arms and non-operative arms of studies. A simple correlation analysis (using Pearson's correlation coefficient) on all SMCs between each pair of time points will be used to assess the relationship between intervals.

If there is sufficient new data in comparison to previous reviews[16], we will conduct a separate analysis comparing outcomes for operative interventions versus non operative interventions. A random effects model will be used to pool the standardised mean differences (SMDs) of comparable groups of studies in a meta-analysis. Examples of comparisons which will be made include 1) APM versus non-operative interventions; 2) Meniscal repair versus non-operative interventions and 3) Meniscal repair versus APM.

As we are not comparing effectiveness of different interventions against one another e.g. surgery vs physiotherapy formal meta-analysis will not be performed.

All analysis will implemented in R[27] (https://www.r-project .org/) using the metafor package[28].

*Extraction plan for qualitative studies*

A prospective data extraction sheet will be designed to collect the following data: author, year of publication, journal, and aim of study, number of participants, data collection methods, theoretical or methodological underpinnings and results.

Analysis and Meta-synthesis



For peer review only - http://bmjopen.bmj.com/site/about/guidelines.xhtml

If sufficient studies are identified, a meta-synthesis will be performed to translate existing evidence into new theory. Meta-synthesis is a process which involves synthesis of findings from qualitative studies, interpreting the results and generating new intepretations[29]. Each included study will be read and reread to produce a table of first order constructs (direct quotes from the participants in each individual study) and second order constructs (author interpretation of the meaning of individual participant quotes). Thematic coding will be used to identify cluster of themes in order to develop third order constructs (Our interpretation of the cluster themes).

**Discussion**

Previous reviews have focussed on comparing the effectiveness of one surgical intervention (arthroscopic meniscectomy or meniscal repair) versus a placebo or non-operative intervention[12,16]. There has been no review reporting the clinical course or natural history of the condition. This review will provide a comprehensive summary of the patient reported outcomes of all patients being managed with a meniscal tear in the literature. Our analysis will provide an insight into the natural history of the condition and the patterns of change in PROMs following operative management. Patient and public involvement in each of the included studies will be also be explored.

There has been no qualitative synthesis in the literature of patient experiences of meniscal tears. This review will explore and summarise the available qualitative evidences on patient experiences of living with a meniscal tear. This will identify if there is an evidence gap which will require the need for further qualitative research in this patient population.

This review will inform future research through developing the understanding of the natural history of patients with a meniscal tear. It will also inform researchers and healthcare providers on patient views and experiences on living with a meniscal tear.

**Patient and public involvement**



For peer review only - http://bmjopen.bmj.com/site/about/guidelines.xhtml

A PPI group of 18 patients with knee osteoarthritis or meniscal tears was set up to discuss this study idea. Input was provided on the importance of patient focussed dissemination of the results. A patient member (DS) reviewed aspects of the manuscript, in particular the study abstract and dissemination plans. A lay summary of the final results of the review will be produced in combination with DS and we will make it freely available via a study twitter page.

**Ethics and dissemination**

Given the study design, no formal ethical approval will be sought to conduct this review. The findings will be disseminated at national and international conferences. The final report will be submitted for peer reviewed publication. A lay summary will be produced and be freely available via the study twitter page.

**Funding**

This protocol represented research funded by the National Institute of Health Research (NIHR) Doctoral Research Fellowship grant awarded to IA, DRF-2018-11-ST2-030. The views expressed in this publication are those of the authors and not necessarily those of the NHS, the National Institute for Health Research or the Department of Health and Social Care.

**Author contributions**

IA: study conception, drafted and reviewed final manuscript

CK: Drafted and reviewed final manuscript

NP: study conception, drafted and reviewed final manuscript

CH: study conception, drafted and reviewed final manuscript

SS: study conception, drafted and reviewed final manuscript

AP: study conception, drafted and reviewed final manuscript

AM: study conception, drafted and reviewed final manuscript

**Competing interests**

All authors declare no competing interests.

**References**



For peer review only - http://bmjopen.bmj.com/site/about/guidelines.xhtml

1. Baker BE, Peckham AC, Pupparo F, Sanborn JC. Review of meniscal injury and associated sports. *Am J Sports Med* 1985; **13**(1): 1-4.
2. Abram SGF, Judge A, Beard DJ, Price AJ. Adverse outcomes after arthroscopic partial meniscectomy: a study of 700 000 procedures in the national Hospital Episode Statistics database for England. *Lancet* 2018; **392**(10160): 2194-202.
3. Hulet C PH, Peretti G, Denti M. Surgery of the Meniscus. 1 ed. Heidelberg New York Dordrecht London: Springer; 2016.
4. Bourne RB, Finlay JB, Papadopoulos P, Andreae P. The effect of medial meniscectomy on strain distribution in the proximal part of the tibia. *J Bone Joint Surg Am* 1984; **66**(9): 1431-7.
5. Freutel M, Seitz AM, Galbusera F, et al. Medial meniscal displacement and strain in three dimensions under compressive loads: MR assessment. *J Magn Reson Imaging* 2014; **40**(5): 1181-8.
6. Abram SGF, Beard DJ, Price AJ. National consensus on the definition, investigation, and classification of meniscal lesions of the knee. *Knee* 2018; **25**(5): 834-40.
7. Poulsen MR, Johnson DL. Meniscal injuries in the young, athletically active patient. *Phys Sportsmed* 2011; **39**(1): 123-30.
8. Rathleff CR, Cavallius C, Jensen HP, et al. Successful conservative treatment of patients with MRI-verified meniscal lesions. *Knee Surg Sports Traumatol Arthrosc* 2015; **23**(1): 178-83.
9. Glyn-Jones S, Palmer AJ, Agricola R, et al. Osteoarthritis. *Lancet* 2015; **386**(9991): 376-87.
10. Uthman OA, van der Windt DA, Jordan JL, et al. Exercise for lower limb osteoarthritis: systematic review incorporating trial sequential analysis and network meta-analysis. *BMJ* 2013; **347**: f5555.
11. Abram SGF, Beard DJ, Price AJ. Arthroscopic meniscal surgery: a national society treatment guideline and consensus statement. *Bone Joint J* 2019; **101-B**(6): 652-9.
12. Thorlund JB, Juhl CB, Roos EM, Lohmander LS. Arthroscopic surgery for degenerative knee: systematic review and meta-analysis of benefits and harms. *Br J Sports Med* 2015; **49**(19): 1229-35.
13. Kise NJ, Risberg MA, Stensrud S, Ranstam J, Engebretsen L, Roos EM. Exercise therapy versus arthroscopic partial meniscectomy for degenerative meniscal tear in middle aged patients: randomised controlled trial with two year follow-up. *BMJ* 2016; **354**: i3740.
14. Sihvonen R, Paavola M, Malmivaara A, et al. Arthroscopic partial meniscectomy versus sham surgery for a degenerative meniscal tear. *N Engl J Med* 2013; **369**(26): 2515-24.
15. Siemieniuk RAC, Harris IA, Agoritsas T, et al. Arthroscopic surgery for degenerative knee arthritis and meniscal tears: a clinical practice guideline. *Br J Sports Med* 2018; **52**(5): 313.
16. Abram SGF, Hopewell S, Monk AP, Bayliss LE, Beard DJ, Price AJ. Arthroscopic partial meniscectomy for meniscal tears of the knee: a systematic review and meta-analysis. *Br J Sports Med* 2019.
17. Pihl K, Ensor J, Peat G, et al. Wild goose chase - no predictable patient subgroups benefit from meniscal surgery: patient-reported outcomes of 641 patients 1 year after surgery. *Br J Sports Med* 2019.
18. Thorlund JB, Englund M, Christensen R, et al. Patient reported outcomes in patients undergoing arthroscopic partial meniscectomy for traumatic or degenerative meniscal tears: comparative prospective cohort study. *BMJ* 2017; **356**: j356.
19. Khatri C, Ahmed I, Parsons H, et al. The Natural History of Full-Thickness Rotator Cuff Tears in Randomized Controlled Trials: A Systematic Review and Meta-analysis. *Am J Sports Med* 2019; **47**(7): 1734-43.
20. Artus M, van der Windt DA, Jordan KP, Hay EM. Low back pain symptoms show a similar pattern of improvement following a wide range of primary care treatments: a systematic review of randomized clinical trials. *Rheumatology (Oxford)* 2010; **49**(12): 2346-56.
21. Analytics C. Endnote X9. EndNote X9.2 ed. Philadephia, USA: Clavirate Analytics; 2018.
22. Green S, Higgins J. Cochrane handbook for systematic reviews of interventions. Version; 2005.



For peer review only - http://bmjopen.bmj.com/site/about/guidelines.xhtml

23. Stang A. Critical evaluation of the Newcastle-Ottawa scale for the assessment of the quality of nonrandomized studies in meta-analyses. *Eur J Epidemiol* 2010; **25**(9): 603-5.
24. programme CAs. CASP systematic review checklist. https://casp-uk.net/wp-content/uploads/2018/01/CASP-Systematic-Review-Checklist_2018.pdf (accessed 02/09/2019 2019).
25. Guyatt GH, Oxman AD, Vist GE, et al. GRADE: an emerging consensus on rating quality of evidence and strength of recommendations. *BMJ* 2008; **336**(7650): 924-6.
26. Morris SB. Estimating Effect Sizes From Pretest-Posttest-Control Group Designs. *Organizational Research Methods* 2008; **11**(2): 364-86.
27. team RC. R: A language and environment for statistical computing. Vienna, Autria: R Foundation for Statistical Computing; 2018.
28. W V. Conducting meta-analyses in R with the metafor package. *Journal of statistical software* 2010; **36**(3): 1-48.
29. Walsh D, Downe S. Meta-synthesis method for qualitative research: a literature review. *J Adv Nurs* 2005; **50**(2): 204-11.

For peer review only

**Appendix**

Appendix 1

Search strategy for quantitative aspect of review

Database: Ovid MEDLINE(R) ALL <1946 to September 17, 2019>

Search Strategy:

--------------------------------------------------------------------------------

1 exp Tibial Meniscus Injuries/ or exp Meniscus/ (8082)

2 (menisc* adj3 (tear* or injur* or damage*)).mp. [mp=title, abstract, original title, name of substance word, subject heading word, floating sub-heading word, keyword heading word, organism supplementary concept word, protocol supplementary concept word, rare disease supplementary concept word, unique identifier, synonyms] (7547)

3 1 or 2 (10876)

4 arthroscopy.mp. or exp Arthroscopy/ (28087)

5 exp Meniscectomy/ or meniscect*.mp. (3054)

6 (menisc* adj3 repair).mp. [mp=title, abstract, original title, name of substance word, subject heading word, floating sub-heading word, keyword heading word, organism supplementary concept



For peer review only - http://bmjopen.bmj.com/site/about/guidelines.xhtml

word, protocol supplementary concept word, rare disease supplementary concept word, unique identifier, synonyms] (1565)

7 4 or 5 or 6 (30599)

8 (random$ or placebo$ or single blind$ or double blind$ or triple blind$).mp. [mp=title, abstract, original title, name of substance word, subject heading word, floating sub-heading word, keyword heading word, organism supplementary concept word, protocol supplementary concept word, rare disease supplementary concept word, unique identifier, synonyms] (1389493)

9 exp Randomized Controlled Trial/ (490118)

10 Controlled Clinical Trial/ (93257)

11 exp Clinical Trials as Topic/ (330202)

12 Comparative Study/ (1839704)

13 exp Cohort Studies/ (1898633)

14 Observational Study/ (67005)

15 Prospective Studies/ (513619)

16 8 or 9 or 10 or 11 or 12 or 13 or 14 or 15 (4611873)

17 3 and 7 and 16 (2235)

***************************

Appendix 2

Search strategy for qualitative aspect of review

Database: Ovid MEDLINE(R) ALL <1946 to September 17, 2019>

Search Strategy:

--------------------------------------------------------------------------------

1 exp Tibial Meniscus Injuries/ or exp Meniscus/ (8082)

2 (menisc* adj3 (tear* or injur* or damage*)).mp. [mp=title, abstract, original title, name of substance word, subject heading word, floating sub-heading word, keyword heading word, organism supplementary concept word, protocol supplementary concept word, rare disease supplementary concept word, unique identifier, synonyms] (7547)

3 1 or 2 (10876)

4 health knowledge, attitudes, practice/ or "patient acceptance of health care"/ or patient satisfaction/ (216398)

5 exp Interview/ (28684)

6 exp "Surveys and Questionnaires"/ (975116)



For peer review only - http://bmjopen.bmj.com/site/about/guidelines.xhtml

For peer review only

7 exp Focus Groups/ (27525)

8 4 or 5 or 6 or 7 (1157450)

9 (patient$ adj3 (view$ or opinion$ or awareness or tolerance or perception or persistenc$ or attitude$ or compliance or satisfaction or concern$ or belief$ or feeling$ or position or idea$ or preference$ or choice$)).mp. [mp=title, abstract, original title, name of substance word, subject heading word, floating sub-heading word, keyword heading word, organism supplementary concept word, protocol supplementary concept word, rare disease supplementary concept word, unique identifier, synonyms] (268702)

10 (Discomfort or comfort or inconvenience or bother$4 or trouble or fear$ or anxiety or anxious or worr$3).tw. (334544)

11 8 or 9 or 10 (1567999)

12 3 and 11 (723)

***************************

For peer review only



For peer review only - http://bmjopen.bmj.com/site/about/guidelines.xhtml

**PRISMA-P (Preferred Reporting Items for Systematic review and Meta-Analysis Protocols) 2015 checklist: recommended items to address in a systematic review protocol***

| Section and topic | Item No | Checklist item |
|---|---|---|
| **ADMINISTRATIVE INFORMATION** | | |
| Title: | | |
| Identification | 1a | Identify the report as a protocol of a systematic review – **Found on title page on title**. |
| Update | 1b | If the protocol is for an update of a previous systematic review, identify as such – **Not applicable** |
| Registration | 2 | If registered, provide the name of the registry (such as PROSPERO) and registration number- **Found on Page 2 abstract** |
| Authors: | | |
| Contact | 3a | Provide name, institutional affiliation, e-mail address of all protocol authors; provide physical mailing address of corresponding author – **Found on Page 1 title page** |
| Contributions | 3b | Describe contributions of protocol authors and identify the guarantor of the review – **Found on page 10 author contributions** |
| Amendments | 4 | If the protocol represents an amendment of a previously completed or published protocol, identify as such and list changes; otherwise, state plan for documenting important protocol amendments – **Not applicable** |
| Support: | | |
| Sources | 5a | Indicate sources of financial or other support for the review –**Found on page 10 Funding** |
| Sponsor | 5b | Provide name for the review funder and/or sponsor – **Found on page 10 Funding** |
| Role of sponsor or funder | 5c | Describe roles of funder(s), sponsor(s), and/or institution(s), if any, in developing the protocol – **Found on page 10 Funding** |
| **INTRODUCTION** | | |
| Rationale | 6 | Describe the rationale for the review in the context of what is already known – **Found on page 4 Introduction** |
| Objectives | 7 | Provide an explicit statement of the question(s) the review will address with reference to participants, interventions, comparators, and outcomes (PICO) – **Found on Page 5 objectives** |
| **METHODS** | | |
| Eligibility criteria | 8 | Specify the study characteristics (such as PICO, study design, setting, time frame) and report characteristics (such as years considered, language, publication status) to be used as criteria for eligibility for the review – **Found on page 5 and 6 Eligibility criteria** |
| Information sources | 9 | Describe all intended information sources (such as electronic databases, contact with study authors, trial registers or other grey literature sources) with planned dates of coverage - **Found on page 6 Search strategy and quality assessment** |
| Search strategy | 10 | Present draft of search strategy to be used for at least one electronic database, including planned limits, such that it could be repeated – **Found on page 12/13 Appendix 1/2** |
| Study records: | | |

For peer review only

| | | |
|---|---|---|
| Data management | 11a | Describe the mechanism(s) that will be used to manage records and data throughout the review **– Found on page 6-8 Search strategy and quality assessment** |
| Selection process | 11b | State the process that will be used for selecting studies (such as two independent reviewers) through each phase of the review (that is, screening, eligibility and inclusion in meta-analysis) – **Found on page 6-8** |
| Data collection process | 11c | Describe planned method of extracting data from reports (such as piloting forms, done independently, in duplicate), any processes for obtaining and confirming data from investigators – **Found on page 7-8 Data extraction** |
| Data items | 12 | List and define all variables for which data will be sought (such as PICO items, funding sources), any pre-planned data assumptions and simplifications - **Found on page 7-8 Data extraction** |
| Outcomes and prioritization | 13 | List and define all outcomes for which data will be sought, including prioritization of main and additional outcomes, with rationale **– Found on page 7-8 Outcomes** |
| Risk of bias in individual studies | 14 | Describe anticipated methods for assessing risk of bias of individual studies, including whether this will be done at the outcome or study level, or both; state how this information will be used in data synthesis – **Found on page 6 quality assessment** |
| Data synthesis | 15a | Describe criteria under which study data will be quantitatively synthesised |
| | 15b | If data are appropriate for quantitative synthesis, describe planned summary measures, methods of handling data and methods of combining data from studies, including any planned exploration of consistency (such as $I^2$, Kendall's $\tau$) |
| | 15c | Describe any proposed additional analyses (such as sensitivity or subgroup analyses, meta-regression) |
| | 15d | If quantitative synthesis is not appropriate, describe the type of summary planned<br><br>**All found on page 7-8 Extraction plan** |
| Meta-bias(es) | 16 | Specify any planned assessment of meta-bias(es) (such as publication bias across studies, selective reporting within studies) – **Not applicable** |
| Confidence in cumulative evidence | 17 | Describe how the strength of the body of evidence will be assessed (such as GRADE) – **Found on page 8 Search strategy and quality assessment** |

*** It is strongly recommended that this checklist be read in conjunction with the PRISMA-P Explanation and Elaboration (cite when available) for important clarification on the items. Amendments to a review protocol should be tracked and dated. The copyright for PRISMA-P (including checklist) is held by the PRISMA-P Group and is distributed under a Creative Commons Attribution Licence 4.0.**

*From: Shamseer L, Moher D, Clarke M, Ghersi D, Liberati A, Petticrew M, Shekelle P, Stewart L, PRISMA-P Group. Preferred reporting items for systematic review and meta-analysis protocols (PRISMA-P) 2015: elaboration and explanation. BMJ. 2015 Jan 2;349(jan02 1):g7647.*

For peer review only

BMJ Open

# The Meniscal tear outcome (METRO) review: A protocol for a systematic review summarising the clinical course and patient experiences of meniscal tears in the current literature.

| | |
|---|---|
| Journal: | *BMJ Open* |
| Manuscript ID | bmjopen-2019-036247.R1 |
| Article Type: | Protocol |
| Date Submitted by the Author: | 30-Mar-2020 |
| Complete List of Authors: | Ahmed, Imran; University of Warwick, Clinical Trials Unit<br>Khatri, Chetan<br>Dhaif, Fatema; University of Warwick, Warwick Medical School<br>Parsons, Nicholas; University of Warwick, Warwick Medical School<br>Hutchinson, Charles; University of Warwick, Warwick Medical School<br>Staniszewska, Sophie; University of Warwick, Warwick Medical School<br>Price, Andrew; University of Oxford, Nuffield Department of Orthopaedics, Rheumatology and Musculoskeletal Sciences<br>Metcalfe, Andrew; University of Warwick, Warwick Clinical trials Unit |
| <b>Primary Subject Heading</b>: | Sports and exercise medicine |
| Secondary Subject Heading: | Surgery |
| Keywords: | Knee < ORTHOPAEDIC & TRAUMA SURGERY, Orthopaedic & trauma surgery < SURGERY, Musculoskeletal disorders < ORTHOPAEDIC & TRAUMA SURGERY |
| | |

SCHOLARONE™
Manuscripts

*I, the Submitting Author has the right to grant and does grant on behalf of all authors of the Work (as defined in the below author licence), an exclusive licence and/or a non-exclusive licence for contributions from authors who are: i) UK Crown employees; ii) where BMJ has agreed a CC-BY licence shall apply, and/or iii) in accordance with the terms applicable for US Federal Government officers or employees acting as part of their official duties; on a worldwide, perpetual, irrevocable, royalty-free basis to BMJ Publishing Group Ltd ("BMJ") its licensees and where the relevant Journal is co-owned by BMJ to the co-owners of the Journal, to publish the Work in this journal and any other BMJ products and to exploit all rights, as set out in our licence.*

*The Submitting Author accepts and understands that any supply made under these terms is made by BMJ to the Submitting Author unless you are acting as an employee on behalf of your employer or a postgraduate student of an affiliated institution which is paying any applicable article publishing charge ("APC") for Open Access articles. Where the Submitting Author wishes to make the Work available on an Open Access basis (and intends to pay the relevant APC), the terms of reuse of such Open Access shall be governed by a Creative Commons licence – details of these licences and which Creative Commons licence will apply to this Work are set out in our licence referred to above.*

*Other than as permitted in any relevant BMJ Author's Self Archiving Policies, I confirm this Work has not been accepted for publication elsewhere, is not being considered for publication elsewhere and does not duplicate material already published. I confirm all authors consent to publication of this Work and authorise the granting of this licence.*

For peer review only

**The Meniscal tear outcome (METRO) review: A protocol for a systematic review summarising the clinical course and patient experiences of meniscal tears in the current literature .**

Imran Ahmed (Imran.ahmed4@nhs.net)

Chetan Khatri (chetan.khatri@gmail.com)

Fatema Ghassan Dhaif (fdhaif13@gmail.com)

Nicholas Parsons (Nick.parsons@warwick.ac.uk)

Charles Hutchinson (c.e.hutchinson@warwick.ac.uk)

Sophie Staniszewska (sophie.staniszewska@warwick.ac.uk)

Andrew Price (Andrew.price@ndorms.ox.ac.uk)

Andrew Metcalfe (a.metcalfe@warwick.ac.uk)

Corresponding Author

Imran Ahmed

Trauma and Orthopaedics

Warwick Clinical trials unit

University Hospital Coventry and Warwickshire

Clifford Bridge Road

Coventry

CV2 2DX

Imran.ahmed4@nhs.net

Orchid ID: 0000-0003-2774-9954

For peer review only

## Abstract

### Introduction

Meniscal tears are a common knee injury with an incidence of 60 per 100,000. Management of meniscal tears can include either non-operative measures or operative procedures such as arthroscopic partial meniscectomy (APM). Despite substantial research evaluating the effectiveness of APM in the recent past, little is known about the clinical course or the experiences of patients with a meniscal tear.

### Aim

To summarise the short to long term patterns of variability in outcome in patients with a meniscal tear.

To summarise the evidence on patient experiences of meniscal tears. In particular focussing on patient experiences of treatment options, treatment pathways and their views of the outcomes used in meniscal tear research.

### Method and analysis

Two search strategies will be developed to identify citations from EMBASE, MEDLINE, AMED, CENTRAL, Web of Science and Sociofile. For the quantitative review we will identify studies reporting patient reported outcomes measures (PROMs) in patients after a meniscal tear. The standardised mean change (SMC) will be used to assess the variation in size of response and summarise the overall response to each treatment option. All studies will undergo quality assessment using either the Cochrane Risk of Bias or the Newcastle-Ottawa tool.

A qualitative systematic review will be used to identify studies reporting views and experiences of patients with a meniscal tear. All studies will be assessed using the Critical Appraisal Skills Programme (CASP) tool and if sufficient data is present a meta-synthesis will be performed to identify first, second and third order constructs.

### Ethics and Dissemination

Given the nature of this study, no formal ethical approval will be sought. Results from the review will be disseminated at national conferences and will be submitted to a peer reviewed journal for publication. Lay summaries will be freely available via the study twitter page.


**Registration:** Prospero ID CRD42019122179

**Word count:** 2853



For peer review only - http://bmjopen.bmj.com/site/about/guidelines.xhtml

**Article summary: Strengths and limitations**

- Comprehensive nature of review including patients undergoing all management options.
- Analysis plan in keeping with previously published systematic reviews
- Risk of publication bias as only English Language studies are to be included
- Patient and public involvement in the dissemination of results

For peer review only



For peer review only - http://bmjopen.bmj.com/site/about/guidelines.xhtml

## Introduction

Meniscal tears are a common knee injury with an incidence of 60-70 per 100,000 and they account for 70,000 hospital admissions per year in the UK[1,2].The menisci fibrocartilaginous structures are situated within the knee[3]. The principal role of the menisci is to distribute compressive load within the knee joint by increasing the contact area and reducing the contact pressure between the tibia and the femur[3-5]. This may play a crucial role in preventing osteoarthritis. Risk factors for meniscal tears include age, gender, body mass index (BMI), presence of degeneration within the knee and level of activity e.g. participation in sport[3]. Meniscal tears can be asymptomatic or present with symptoms such as knee pain, swelling, stiffness or locking of the knee[3,6,7], however, it is unknown if these symptoms are caused by the meniscal tear specifically.

The management options for treatment of meniscal tears include watchful waiting (observation), medical therapy (intra-artciular injections, analgesia), exercise therapy and surgery[8-11]. Recent evidence has questioned the effectiveness of surgery when compared to exercise therapy or other non-operative interventions[12-14]. Despite this evidence, the number of arthroscopies has almost doubled over the last 15 years[15,16]. Many surgeons and researchers believe surgery may be beneficial in a subset of patients and over recent years there has been substantial growth in the number of studies and systematic reviews in the management of meniscal tears[17-19]. These studies are yet to identify a clear superior treatment option in this patient population, or to identify a true subset that may benefit from surgery.

Research into other musculoskeletal conditions has demonstrated that outcomes steadily improve over time regardless of treatment[20,21]. This could be due to the natural history of the condition or simply regression to the mean. The natural history of a condition is a crucial factor to consider when interpretation of outcomes from interventions of patients with meniscal tears. Prospective studies with well-defined entry criteria and follow up points provide an excellent source of information on the natural history of a condition[20]. Researchers and clinicians need to better understand the natural history of meniscal tears to plan treatment decisions and future research.

In addition to understanding the clinical course of patients being managed with a meniscal tears, we need to understand patient views and experiences of managing meniscal tears as a form of patient based evidence which we utilise alongside clinical and economic forms of evidence. Recent treatment guidelines were developed, based on the consensus of 25 international experts, by the British Association for Surgery of the Knee (BASK)[11]. However, no patients were involved in the development of these guidelines. In order to treat meniscal tears more effectively and plan



For peer review only - http://bmjopen.bmj.com/site/about/guidelines.xhtml

informative research we need to understand patient views and expectations in the treatment of meniscal tears.

**Objectives**

**Quantitative review**

The objective of the quantitative review is to summarise the short, medium and long term temporal patterns of change in patient reported outcome measures (PROMs) in patients with a meniscal tear regardless of treatment .

**Qualitative review**

The objective of the qualitative review is to identify and summarise the evidence on patient experiences of meniscal tears. In particular we will focus on patient experiences of treatment options, treatment pathways and their views of the outcomes used in meniscal tear research.

## Methods and analysis

This study protocol was developed in accordance with the PRISMA-P (Preferred Reporting Items for Systematic review and Meta-analysis Protocols) checklist. In addition to this manuscript the protocol has been registered on the PROSPERO database and can be found at https://www.crd.york.ac.uk/PROSPERO/display_record.php?RecordID=122179

**Eligibility criteria**

Quantitative review

Inclusion criteria

- Study design
    - Full text Randomised controlled trials in patients treated for a meniscal tear (for example, comparing surgery or a non-operative intervention (exercise therapy, pharmacological therapy or observation) versus a comparator group).
    - Prospective cohort studies adjusted for case-mix reporting outcomes in patients being treated for a meniscal tear.
- Studies reporting an established knee related PROMs score for at least three months.



For peer review only - http://bmjopen.bmj.com/site/about/guidelines.xhtml

For peer review only

- English language studies only.

Exclusion criteria

- Studies not reporting any of the clinical outcomes selected for this review.
- Studies reporting outcomes in patients with additional major knee ligament injury or fractures around the knee.
- Abstract or conference publications.

Qualitative review

Inclusion criteria

- Qualitative studies reporting the views and experiences of patients with a meniscal tear, implementing all types of qualitative methodology.
- English language studies only.

Exclusion criteria

- Abstract or conference publications

**Search Strategy and Quality assessment**

Following consultation with a librarian a search strategy was devised (see supplementary file). The authors aim to search the following databases MEDLINE; Excerpta Medica Database (EMBASE); Allied and Complementary Medicine (AMED); and Cochrane Central Register of Controlled Trials (CENTRAL) using OVID Sp. We will also search Web of science and sociofile for qualitative studies. Search strategies can be seen in the Supplementary files (Appendix 1 and 2). We will also search reference lists of included studies to identify further citations. All citations will be imported into Endnote X9 reference management software[22] (Clarivate Analytics, Philadelphia, USA). After removal of duplicates, titles and abstracts will be screened according to the inclusion criteria. The full texts of studies potentially meeting the inclusion criteria will be screened by two authors (IA and CK). These authors will independently assess each study and any discrepancies will be addressed by discussion with a senior author (NP or AM).

RCTs will be qualitatively assessed using the Cochrane risk of bias tool[23]. Observational studies will be assessed using the Newcastle-Ottawa scale[24]. Two authors (IA and CK) will independently assess the quality of each included study with discrepancies being resolved following discussion with a senior author (NP or AM).



For peer review only - http://bmjopen.bmj.com/site/about/guidelines.xhtml

Each qualitative study will be critically appraised using the Critical Appraisal Skills Programme (CASP) for qualitative studies[25]. Each study will be graded as adequate, partially adequate or inadequate. If rated as inadequate the study will be excluded from the meta-synthesis.

Two authors (IA and CK) will independently assess the quality of evidence. We will use the GRADE considerations (study limitations, consistence of effect, imprecision, indirectness and publication bias) to assess the quality of the body of evidence[26]. Decisions to upgrade or downgrade body of evidence will be clearly stated.

**Outcomes**

Primary outcome

The primary outcome measure is knee pain or function measured by at least one of the following validated knee questionnaires : (i) Knee injury and osteoarthritis outcome score (KOOS); (ii) Oxford knee score (OKS); (iii) Western Ontario meniscal evaluation tool (WOMET); (iv) Lysholm score and (v) International Knee Documentation Committee score (IKDC); Western Ontario and McMaster Universities Osteoarthritis Index (WOMAC) score.

**Data extraction**

*Extraction plan for quantitative studies*

For each study we will collect number of patients (in each arm if RCT or non-randomised study) and the intervention type, defined as meniscectomy, meniscal repair, placebo surgery or non-operative. In addition, mean age, gender, BMI, method of diagnosis of tear, imaging findings (presence of OA), patient reported symptoms, whether patient and public involvement (PPI) was reported and means and standard deviations (SDs) of the PROMs score at each time point reported will also be collected. We will also extract information on whether any qualitative data were included in the final study report. If a study does not report mean or SD, we will contact the study authors for further information. If the SD is not provided then where possible estimates will be calculated from other reported statistics (data summaries), e.g. a test statistic or P value, using standard methods as described in the Cochrane handbook[23]. If data are not represented in a simple numeric format (e.g. in a table or in the study text), but are presented graphically only (e.g. a figure or graph), two authors (IA and CK) will extract the data manually. If failing this we are unable to extract the data we will contact the study authors and request the data.



For peer review only - http://bmjopen.bmj.com/site/about/guidelines.xhtml

Data analysis

Outcome scores will be plotted against time from study intervention (e.g. non-operative (exercise therapy or pharmacological therapy) or surgery) to describe change from baseline (at the point of recruitment) to all follow up time points reported in the treatment arms included. This will provide a simple visual representation of the clinical course of the condition. If studies report a observation or a 'watch and wait' arm where no treatment is initiated we will plot this separately. This will provide an insight into the natural history of the condition.

To determine variation in size of response, the change of outcome will be assessed by calculating the bias-corrected standardised mean change (SMC) at 3, 6, 12, 24, 36, 48 and 60 months . The SMC is calculated by subtracting the follow-up mean score for the PROM of interest from the baseline mean score. This is then divided by the pooled SD and multiplied by the bias correction factor based on group size. Follow up or baseline SD will be used if the pooled SD is not reported or provided when study authors are contacted for further information. The 95% confidence intervals (CI) will be calculated using estimates of the variance of the SMC[27].

Studies using a variety of outcomes scales or PROMs can be pooled as the SMC standardises the measurement of change. As in previously reported meta-analyses we will calculate a combined pooled SMC for each time point using a random effects model[20,21]. We will do this for the meniscectomy arm, meniscal repair arms and non-operative arms of studies. A simple correlation analysis (using Pearson's correlation coefficient) on all SMCs between each pair of time points will be used to assess the relationship between intervals.

If there is sufficient new data in comparison to previous reviews[17], we will conduct a separate analysis comparing outcomes for operative interventions versus non operative interventions. A random effects model will be used to pool the standardised mean differences (SMDs) of comparable groups of studies in a meta-analysis. Examples of comparisons which will be made include 1) APM versus non-operative interventions; 2) Meniscal repair versus non-operative interventions and 3) Meniscal repair versus APM.

As we are not comparing effectiveness of different interventions against one another e.g. surgery vs exercise therapy formal meta-analysis will not be performed.

All analysis will implemented in R[28] (https://www.r-project .org/) using the metafor package[29].

*Extraction plan for qualitative studies*



For peer review only - http://bmjopen.bmj.com/site/about/guidelines.xhtml

A prospective data extraction sheet will be designed to collect the following data: author, year of publication, journal, and aim of study, number of participants, data collection methods, theoretical or methodological underpinnings and results.

Analysis and Meta-synthesis

If sufficient studies are identified, a meta-synthesis will be performed to translate existing evidence into new theory. Meta-synthesis is a process which involves synthesis of findings from qualitative studies, interpreting the results and generating new intepretations[30]. Each included study will be read and reread to produce a table of first order constructs (direct quotes from the participants in each individual study) and second order constructs (author interpretation of the meaning of individual participant quotes). We will collect data (or quotes) on participant views on treatment options, treatment pathways and the current outcomes used in meniscal tear research. Thematic coding will be used to identify cluster of themes in order to develop third order constructs (Our interpretation of the cluster themes).

**Discussion**

Previous reviews have focussed on comparing the effectiveness of one surgical intervention (arthroscopic meniscectomy or meniscal repair) versus a placebo or non-operative intervention[12,17]. There has been no review reporting the clinical course or natural history of the condition. This review will provide a comprehensive summary of the patient reported outcomes of all patients being managed with a meniscal tear in the literature. Our analysis will provide an insight into the natural history of the condition and the patterns of change in PROMs following operative management. Patient and public involvement in each of the included studies will be also be explored.

There has been no qualitative synthesis in the literature of patient experiences of meniscal tears. This review will explore and summarise the available qualitative evidences on patient experiences of living with a meniscal tear. This will identify if there is an evidence gap which will require the need for further qualitative research in this patient population.



For peer review only - http://bmjopen.bmj.com/site/about/guidelines.xhtml

This review will inform future research through developing the understanding of the natural history of patients with a meniscal tear. It will also inform researchers and healthcare providers on patient views and experiences on living with a meniscal tear.

A potential weakness of this review is based on the studies included. Studies may not include an observation arm where there is no treatment initiated. As a result it will be difficult to describe the true natural history of patients with meniscal tears. Another weakness is that we are pooling the results of all patients with meniscal tears where there is the possibility of clinical heterogeneity in the sample. A recent cohort study reported factors such as age, gender and symptom duration did not influence treatment outcome[18]. As a result a decision was taken to group all tears together.

Strengths of the review include its comprehensive nature of its design. We will include patients being managed by both operative and non-operative measures. Studies will be quality assessed using appropriate methodology and the body of evidence will be assessed using the GRADE criteria. A futher strength of this piece of work is the incorporation of qualitative data. This will be the first review to summarise the evidence exploring participant views on treatment options and treatment pathways. This will provide further insight for clinicians in order to determine treatment decisions.

## Patient and public involvement

A PPI group of 18 patients with knee osteoarthritis or meniscal tears was set up to discuss this study idea. Input was provided on the importance of patient focussed dissemination of the results. A patient member (DS) reviewed aspects of the manuscript, in particular the study abstract and dissemination plans. A lay summary of the final results of the review will be produced in combination with DS and we will make it freely available via a study twitter page.

## Ethics and dissemination

Given the study design, no formal ethical approval will be sought to conduct this review. The findings will be disseminated at national and international conferences. The final report will be submitted for



For peer review only - http://bmjopen.bmj.com/site/about/guidelines.xhtml

peer reviewed publication. A lay summary will be produced and be freely available via the study twitter page.

**Funding**

This protocol represented research funded by the National Institute of Health Research (NIHR) Doctoral Research Fellowship grant awarded to IA, DRF-2018-11-ST2-030. The views expressed in this publication are those of the authors and not necessarily those of the NHS, the National Institute for Health Research or the Department of Health and Social Care.

**Author contributions**

IA: study conception, drafted and reviewed final manuscript

CK: Drafted and reviewed final manuscript

FD: Revisions to manuscript and reviewed final manuscript

NP: study conception, drafted and reviewed final manuscript

CH: study conception, drafted and reviewed final manuscript

SS: study conception, drafted and reviewed final manuscript

AP: study conception, drafted and reviewed final manuscript

AM: study conception, drafted and reviewed final manuscript

**Competing interests**

All authors declare no competing interests.

**References**

1. Baker BE, Peckham AC, Pupparo F, Sanborn JC. Review of meniscal injury and associated sports. *Am J Sports Med* 1985; **13**(1): 1-4.
2. Abram SGF, Judge A, Beard DJ, Price AJ. Adverse outcomes after arthroscopic partial meniscectomy: a study of 700 000 procedures in the national Hospital Episode Statistics database for England. *Lancet* 2018; **392**(10160): 2194-202.
3. Hulet C PH, Peretti G, Denti M. Surgery of the Meniscus. 1 ed. Heidelberg New York Dordrecht London: Springer; 2016.
4. Bourne RB, Finlay JB, Papadopoulos P, Andreae P. The effect of medial meniscectomy on strain distribution in the proximal part of the tibia. *J Bone Joint Surg Am* 1984; **66**(9): 1431-7.
5. Freutel M, Seitz AM, Galbusera F, et al. Medial meniscal displacement and strain in three dimensions under compressive loads: MR assessment. *J Magn Reson Imaging* 2014; **40**(5): 1181-8.
6. Abram SGF, Beard DJ, Price AJ. National consensus on the definition, investigation, and classification of meniscal lesions of the knee. *Knee* 2018; **25**(5): 834-40.



For peer review only - http://bmjopen.bmj.com/site/about/guidelines.xhtml

7. Poulsen MR, Johnson DL. Meniscal injuries in the young, athletically active patient. *Phys Sportsmed* 2011; **39**(1): 123-30.
8. Rathleff CR, Cavallius C, Jensen HP, et al. Successful conservative treatment of patients with MRI-verified meniscal lesions. *Knee Surg Sports Traumatol Arthrosc* 2015; **23**(1): 178-83.
9. Glyn-Jones S, Palmer AJ, Agricola R, et al. Osteoarthritis. *Lancet* 2015; **386**(9991): 376-87.
10. Uthman OA, van der Windt DA, Jordan JL, et al. Exercise for lower limb osteoarthritis: systematic review incorporating trial sequential analysis and network meta-analysis. *BMJ* 2013; **347**: f5555.
11. Abram SGF, Beard DJ, Price AJ. Arthroscopic meniscal surgery: a national society treatment guideline and consensus statement. *Bone Joint J* 2019; **101-B**(6): 652-9.
12. Thorlund JB, Juhl CB, Roos EM, Lohmander LS. Arthroscopic surgery for degenerative knee: systematic review and meta-analysis of benefits and harms. *Br J Sports Med* 2015; **49**(19): 1229-35.
13. Kise NJ, Risberg MA, Stensrud S, Ranstam J, Engebretsen L, Roos EM. Exercise therapy versus arthroscopic partial meniscectomy for degenerative meniscal tear in middle aged patients: randomised controlled trial with two year follow-up. *BMJ* 2016; **354**: i3740.
14. Sihvonen R, Paavola M, Malmivaara A, et al. Arthroscopic partial meniscectomy versus sham surgery for a degenerative meniscal tear. *N Engl J Med* 2013; **369**(26): 2515-24.
15. Siemieniuk RAC, Harris IA, Agoritsas T, et al. Arthroscopic surgery for degenerative knee arthritis and meniscal tears: a clinical practice guideline. *Br J Sports Med* 2018; **52**(5): 313.
16. Abram SGF, Judge A, Beard DJ, Wilson HA, Price AJ. Temporal trends and regional variation in the rate of arthroscopic knee surgery in England: analysis of over 1.7 million procedures between 1997 and 2017. Has practice changed in response to new evidence? *Br J Sports Med* 2019; **53**(24): 1533-8.
17. Abram SGF, Hopewell S, Monk AP, Bayliss LE, Beard DJ, Price AJ. Arthroscopic partial meniscectomy for meniscal tears of the knee: a systematic review and meta-analysis. *Br J Sports Med* 2019.
18. Pihl K, Ensor J, Peat G, et al. Wild goose chase - no predictable patient subgroups benefit from meniscal surgery: patient-reported outcomes of 641 patients 1 year after surgery. *Br J Sports Med* 2019.
19. Thorlund JB, Englund M, Christensen R, et al. Patient reported outcomes in patients undergoing arthroscopic partial meniscectomy for traumatic or degenerative meniscal tears: comparative prospective cohort study. *BMJ* 2017; **356**: j356.
20. Khatri C, Ahmed I, Parsons H, et al. The Natural History of Full-Thickness Rotator Cuff Tears in Randomized Controlled Trials: A Systematic Review and Meta-analysis. *Am J Sports Med* 2019; **47**(7): 1734-43.
21. Artus M, van der Windt DA, Jordan KP, Hay EM. Low back pain symptoms show a similar pattern of improvement following a wide range of primary care treatments: a systematic review of randomized clinical trials. *Rheumatology (Oxford)* 2010; **49**(12): 2346-56.
22. Analytics C. Endnote X9. EndNote X9.2 ed. Philadephia, USA: Clavirate Analytics; 2018.
23. Green S, Higgins J. Cochrane handbook for systematic reviews of interventions. Version; 2005.
24. Stang A. Critical evaluation of the Newcastle-Ottawa scale for the assessment of the quality of nonrandomized studies in meta-analyses. *Eur J Epidemiol* 2010; **25**(9): 603-5.
25. programme CAs. CASP systematic review checklist. https://casp-uk.net/wp-content/uploads/2018/01/CASP-Systematic-Review-Checklist_2018.pdf (accessed 02/09/2019 2019).
26. Guyatt GH, Oxman AD, Vist GE, et al. GRADE: an emerging consensus on rating quality of evidence and strength of recommendations. *BMJ* 2008; **336**(7650): 924-6.
27. Morris SB. Estimating Effect Sizes From Pretest-Posttest-Control Group Designs. *Organizational Research Methods* 2008; **11**(2): 364-86.

For peer review only



For peer review only - http://bmjopen.bmj.com/site/about/guidelines.xhtml

28. team RC. R: A language and environment for statistical computing. Vienna, Autria: R Foundation for Statistical Computing; 2018.
29. W V. Conducting meta-analyses in R with the metafor package. *Journal of statistical software* 2010; **36**(3): 1-48.
30. Walsh D, Downe S. Meta-synthesis method for qualitative research: a literature review. *J Adv Nurs* 2005; **50**(2): 204-11.

For peer review only

**Supplementary file**

**Appendix 1**

Search strategy for quantitative aspect of review

Database: Ovid MEDLINE(R) ALL <1946 to September 17, 2019>

Search Strategy:

--------------------------------------------------------------------------------

1 exp Tibial Meniscus Injuries/ or exp Meniscus/ (8082)

2 (menisc* adj3 (tear* or injur* or damage*)).mp. [mp=title, abstract, original title, name of substance word, subject heading word, floating sub-heading word, keyword heading word, organism supplementary concept word, protocol supplementary concept word, rare disease supplementary concept word, unique identifier, synonyms] (7547)

3 1 or 2 (10876)

4 arthroscopy.mp. or exp Arthroscopy/ (28087)

5 exp Meniscectomy/ or meniscect*.mp. (3054)

6 (menisc* adj3 repair).mp. [mp=title, abstract, original title, name of substance word, subject heading word, floating sub-heading word, keyword heading word, organism supplementary concept word, protocol supplementary concept word, rare disease supplementary concept word, unique identifier, synonyms] (1565)

7 4 or 5 or 6 (30599)

8 (random$ or placebo$ or single blind$ or double blind$ or triple blind$).mp. [mp=title, abstract, original title, name of substance word, subject heading word, floating sub-heading word, keyword heading word, organism supplementary concept word, protocol supplementary concept word, rare disease supplementary concept word, unique identifier, synonyms] (1389493)

9 exp Randomized Controlled Trial/ (490118)

10 Controlled Clinical Trial/ (93257)

11 exp Clinical Trials as Topic/ (330202)

12 Comparative Study/ (1839704)

13 exp Cohort Studies/ (1898633)

14 Observational Study/ (67005)

15 Prospective Studies/ (513619)

16 8 or 9 or 10 or 11 or 12 or 13 or 14 or 15 (4611873)

17 3 and 7 and 16 (2235)

***************************

For peer review only

**Appendix 2**

Search strategy for qualitative aspect of review

Database: Ovid MEDLINE(R) ALL <1946 to September 17, 2019>

Search Strategy:

--------------------------------------------------------------------------------

1 exp Tibial Meniscus Injuries/ or exp Meniscus/ (8082)

2 (menisc* adj3 (tear* or injur* or damage*)).mp. [mp=title, abstract, original title, name of substance word, subject heading word, floating sub-heading word, keyword heading word, organism supplementary concept word, protocol supplementary concept word, rare disease supplementary concept word, unique identifier, synonyms] (7547)

3 1 or 2 (10876)

4 health knowledge, attitudes, practice/ or "patient acceptance of health care"/ or patient satisfaction/ (216398)

5 exp Interview/ (28684)

6 exp "Surveys and Questionnaires"/ (975116)

7 exp Focus Groups/ (27525)

8 4 or 5 or 6 or 7 (1157450)

9 (patient$ adj3 (view$ or opinion$ or awareness or tolerance or perception or persistenc$ or attitude$ or compliance or satisfaction or concern$ or belief$ or feeling$ or position or idea$ or preference$ or choice$)).mp. [mp=title, abstract, original title, name of substance word, subject heading word, floating sub-heading word, keyword heading word, organism supplementary concept word, protocol supplementary concept word, rare disease supplementary concept word, unique identifier, synonyms] (268702)

10 (Discomfort or comfort or inconvenience or bother$4 or trouble or fear$ or anxiety or anxious or worr$3).tw. (334544)

11 8 or 9 or 10 (1567999)

12 3 and 11 (723)

***************************

For peer review only

**PRISMA-P (Preferred Reporting Items for Systematic review and Meta-Analysis Protocols) 2015 checklist: recommended items to address in a systematic review protocol***

| Section and topic | Item No | Checklist item |
|---|---|---|
| **ADMINISTRATIVE INFORMATION** | | |
| Title: | | |
| Identification | 1a | Identify the report as a protocol of a systematic review – **Found on title page on title**. |
| Update | 1b | If the protocol is for an update of a previous systematic review, identify as such – **Not applicable** |
| Registration | 2 | If registered, provide the name of the registry (such as PROSPERO) and registration number- **Found on Page 2 abstract** |
| Authors: | | |
| Contact | 3a | Provide name, institutional affiliation, e-mail address of all protocol authors; provide physical mailing address of corresponding author – **Found on Page 1 title page** |
| Contributions | 3b | Describe contributions of protocol authors and identify the guarantor of the review – **Found on page 10 author contributions** |
| Amendments | 4 | If the protocol represents an amendment of a previously completed or published protocol, identify as such and list changes; otherwise, state plan for documenting important protocol amendments – **Not applicable** |
| Support: | | |
| Sources | 5a | Indicate sources of financial or other support for the review –**Found on page 10 Funding** |
| Sponsor | 5b | Provide name for the review funder and/or sponsor – **Found on page 10 Funding** |
| Role of sponsor or funder | 5c | Describe roles of funder(s), sponsor(s), and/or institution(s), if any, in developing the protocol – **Found on page 10 Funding** |
| **INTRODUCTION** | | |
| Rationale | 6 | Describe the rationale for the review in the context of what is already known – **Found on page 4 Introduction** |
| Objectives | 7 | Provide an explicit statement of the question(s) the review will address with reference to participants, interventions, comparators, and outcomes (PICO) – **Found on Page 5 objectives** |
| **METHODS** | | |
| Eligibility criteria | 8 | Specify the study characteristics (such as PICO, study design, setting, time frame) and report characteristics (such as years considered, language, publication status) to be used as criteria for eligibility for the review – **Found on page 5 and 6 Eligibility criteria** |
| Information sources | 9 | Describe all intended information sources (such as electronic databases, contact with study authors, trial registers or other grey literature sources) with planned dates of coverage - **Found on page 6 Search strategy and quality assessment** |
| Search strategy | 10 | Present draft of search strategy to be used for at least one electronic database, including planned limits, such that it could be repeated – **Found on page 12/13 Appendix 1/2** |
| Study records: | | |

For peer review only

| | | |
|---|---|---|
| Data management | 11a | Describe the mechanism(s) that will be used to manage records and data throughout the review **– Found on page 6-8 Search strategy and quality assessment** |
| Selection process | 11b | State the process that will be used for selecting studies (such as two independent reviewers) through each phase of the review (that is, screening, eligibility and inclusion in meta-analysis) – **Found on page 6-8** |
| Data collection process | 11c | Describe planned method of extracting data from reports (such as piloting forms, done independently, in duplicate), any processes for obtaining and confirming data from investigators – **Found on page 7-8 Data extraction** |
| Data items | 12 | List and define all variables for which data will be sought (such as PICO items, funding sources), any pre-planned data assumptions and simplifications - **Found on page 7-8 Data extraction** |
| Outcomes and prioritization | 13 | List and define all outcomes for which data will be sought, including prioritization of main and additional outcomes, with rationale **– Found on page 7-8 Outcomes** |
| Risk of bias in individual studies | 14 | Describe anticipated methods for assessing risk of bias of individual studies, including whether this will be done at the outcome or study level, or both; state how this information will be used in data synthesis – **Found on page 6 quality assessment** |
| Data synthesis | 15a | Describe criteria under which study data will be quantitatively synthesised |
| | 15b | If data are appropriate for quantitative synthesis, describe planned summary measures, methods of handling data and methods of combining data from studies, including any planned exploration of consistency (such as $I^2$, Kendall's $\tau$) |
| | 15c | Describe any proposed additional analyses (such as sensitivity or subgroup analyses, meta-regression) |
| | 15d | If quantitative synthesis is not appropriate, describe the type of summary planned<br><br>**All found on page 7-8 Extraction plan** |
| Meta-bias(es) | 16 | Specify any planned assessment of meta-bias(es) (such as publication bias across studies, selective reporting within studies) – **Not applicable** |
| Confidence in cumulative evidence | 17 | Describe how the strength of the body of evidence will be assessed (such as GRADE) – **Found on page 8 Search strategy and quality assessment** |

*** It is strongly recommended that this checklist be read in conjunction with the PRISMA-P Explanation and Elaboration (cite when available) for important clarification on the items. Amendments to a review protocol should be tracked and dated. The copyright for PRISMA-P (including checklist) is held by the PRISMA-P Group and is distributed under a Creative Commons Attribution Licence 4.0.**

*From: Shamseer L, Moher D, Clarke M, Ghersi D, Liberati A, Petticrew M, Shekelle P, Stewart L, PRISMA-P Group. Preferred reporting items for systematic review and meta-analysis protocols (PRISMA-P) 2015: elaboration and explanation. BMJ. 2015 Jan 2;349(jan02 1):g7647.*

For peer review only

BMJ Open

# The Meniscal tear outcome (METRO) review: A protocol for a systematic review summarising the clinical course and patient experiences of meniscal tears in the current literature.

| | |
|---|---|
| Journal: | *BMJ Open* |
| Manuscript ID | bmjopen-2019-036247.R2 |
| Article Type: | Protocol |
| Date Submitted by the Author: | 25-May-2020 |
| Complete List of Authors: | Ahmed, Imran; University of Warwick, Clinical Trials Unit<br>Khatri, Chetan<br>Parsons, Nicholas; University of Warwick, Warwick Medical School<br>Hutchinson, Charles; University of Warwick, Warwick Medical School<br>Staniszewska, Sophie; University of Warwick, Warwick Medical School<br>Price, Andrew; University of Oxford, Nuffield Department of Orthopaedics, Rheumatology and Musculoskeletal Sciences<br>Metcalfe, Andrew; University of Warwick, Warwick Clinical trials Unit |
| <b>Primary Subject Heading</b>: | Sports and exercise medicine |
| Secondary Subject Heading: | Surgery |
| Keywords: | Knee < ORTHOPAEDIC & TRAUMA SURGERY, Orthopaedic & trauma surgery < SURGERY, Musculoskeletal disorders < ORTHOPAEDIC & TRAUMA SURGERY |
| | |

SCHOLARONE™
Manuscripts

*I, the Submitting Author has the right to grant and does grant on behalf of all authors of the Work (as defined in the below author licence), an exclusive licence and/or a non-exclusive licence for contributions from authors who are: i) UK Crown employees; ii) where BMJ has agreed a CC-BY licence shall apply, and/or iii) in accordance with the terms applicable for US Federal Government officers or employees acting as part of their official duties; on a worldwide, perpetual, irrevocable, royalty-free basis to BMJ Publishing Group Ltd ("BMJ") its licensees and where the relevant Journal is co-owned by BMJ to the co-owners of the Journal, to publish the Work in this journal and any other BMJ products and to exploit all rights, as set out in our licence.*

*The Submitting Author accepts and understands that any supply made under these terms is made by BMJ to the Submitting Author unless you are acting as an employee on behalf of your employer or a postgraduate student of an affiliated institution which is paying any applicable article publishing charge ("APC") for Open Access articles. Where the Submitting Author wishes to make the Work available on an Open Access basis (and intends to pay the relevant APC), the terms of reuse of such Open Access shall be governed by a Creative Commons licence – details of these licences and which Creative Commons licence will apply to this Work are set out in our licence referred to above.*

*Other than as permitted in any relevant BMJ Author's Self Archiving Policies, I confirm this Work has not been accepted for publication elsewhere, is not being considered for publication elsewhere and does not duplicate material already published. I confirm all authors consent to publication of this Work and authorise the granting of this licence.*

For peer review only

# The Meniscal tear outcome (METRO) review: A protocol for a systematic review summarising the clinical course and patient experiences of meniscal tears in the current literature.

Imran Ahmed (Imran.ahmed4@nhs.net)

Chetan Khatri (chetan.khatri@gmail.com)

Nicholas Parsons (Nick.parsons@warwick.ac.uk)

Charles Hutchinson (c.e.hutchinson@warwick.ac.uk)

Sophie Staniszewska (sophie.staniszewska@warwick.ac.uk)

Andrew Price (Andrew.price@ndorms.ox.ac.uk)

Andrew Metcalfe (a.metcalfe@warwick.ac.uk)

Corresponding Author

Imran Ahmed

Trauma and Orthopaedics

Warwick Clinical trials unit

University Hospital Coventry and Warwickshire

Clifford Bridge Road

Coventry

CV2 2DX

Imran.ahmed4@nhs.net

Orchid ID: 0000-0003-2774-9954

For peer review only

## Abstract

### Introduction

Meniscal tears are a common knee injury with an incidence of 60 per 100,000. Management of meniscal tears can include either non-operative measures or operative procedures such as arthroscopic partial meniscectomy (APM). Despite substantial research evaluating the effectiveness of APM in the recent past, little is known about the clinical course or the experiences of patients with a meniscal tear.

### Aim

To summarise the short to long term patterns of variability in outcome in patients with a meniscal tear.

To summarise the evidence on patient experiences of meniscal tears. In particular focusing on patient experiences of treatment options, treatment pathways and their views of the outcomes used in meniscal tear research.

### Method and analysis

Two search strategies will be developed to identify citations from EMBASE, MEDLINE, AMED, CENTRAL, Web of Science and Sociofile. The date of our planned search is 14/08/2020. For the quantitative review we will identify studies reporting patient reported outcomes measures (PROMs) in patients after a meniscal tear. The standardised mean change (SMC) will be used to assess the variation in size of response and summarise the overall response to each treatment option. All studies will undergo quality assessment using either the Cochrane Risk of Bias or the Newcastle-Ottawa tool.

A qualitative systematic review will be used to identify studies reporting views and experiences of patients with a meniscal tear. All studies will be assessed using the Critical Appraisal Skills Programme (CASP) tool and if sufficient data is present a meta-synthesis will be performed to identify first, second and third order constructs.

### Ethics and Dissemination

Given the nature of this study, no formal ethical approval will be sought. Results from the review will be disseminated at national conferences and will be submitted to a peer reviewed journal for publication. Lay summaries will be freely available via the study twitter page.

**Registration:** Prospero ID CRD42019122179




For peer review only - http://bmjopen.bmj.com/site/about/guidelines.xhtml

**Word count:** 3018

**Article summary: Strengths and limitations**

- Comprehensive nature of review including patients undergoing all management options.
- Analysis plan in keeping with previously published systematic reviews
- Risk of publication bias as only English Language studies are to be included
- Patient and public involvement from study conception through to planned dissemination of results.

For peer review only



For peer review only - http://bmjopen.bmj.com/site/about/guidelines.xhtml

## Introduction

Meniscal tears are a common knee injury with an annual incidence of 60-70 per 100,000 and they account for 70,000 hospital admissions per year in the UK[1,2].The menisci fibrocartilaginous structures are situated within the knee[3]. The principal role of the menisci is to distribute compressive load within the knee joint by increasing the contact area and reducing the contact pressure between the tibia and the femur[3-5]. This may play a crucial role in preventing osteoarthritis. Risk factors for meniscal tears include age, gender, body mass index (BMI), presence of degeneration within the knee and level of activity e.g. participation in sport[3]. Meniscal tears can be asymptomatic or present with symptoms such as knee pain, swelling, stiffness or locking of the knee[3,6,7], however, it is unknown if these symptoms are caused by the meniscal tear specifically.

The management options for treatment of meniscal tears include watchful waiting (observation), medical therapy (intra-articular injections, analgesia), exercise therapy and surgery[8-11]. Recent evidence has questioned the effectiveness of surgery when compared to exercise therapy or other non-operative interventions[12-14]. Despite this evidence, the number of arthroscopies has almost doubled over the last 15 years[15,16]. Many surgeons and researchers believe surgery may be beneficial in a subset of patients and over recent years there has been substantial growth in the number of studies and systematic reviews in the management of meniscal tears[17-19]. These studies are yet to identify a clear superior treatment option in this patient population, or to identify a true subset that may benefit from surgery.

Research into other musculoskeletal conditions has demonstrated that outcomes steadily improve over time regardless of treatment[20,21]. This could be due to the natural history of the condition or simply regression to the mean. The natural history of a condition is a crucial factor to consider when interpretation of outcomes from interventions of patients with meniscal tears. Prospective studies with well-defined entry criteria and follow-up points provide an excellent source of information on the natural history of a condition[20]. Researchers and clinicians need to better understand the natural history of meniscal tears to plan treatment decisions and future research.

In addition to understanding the clinical course of patients being managed with a meniscal tear, we need to understand patient views and experiences of having and being treated for meniscal tears, as a form of patient based evidence which we utilise alongside clinical and economic forms of evidence[22]. Recent treatment guidelines were developed, based on the consensus of 25



For peer review only - http://bmjopen.bmj.com/site/about/guidelines.xhtml

international experts, by the British Association for Surgery of the Knee (BASK)[11]. However, no patients were involved in the development of these guidelines. In order to treat meniscal tears more effectively and plan informative research we need to understand patient views and expectations in the treatment of meniscal tears.

**Objectives**

**Quantitative review**

The objective of the quantitative review is to summarise the short, medium and long term temporal patterns of change in patient reported outcome measures (PROMs) in patients with a meniscal tear regardless of treatment.

**Qualitative review**

The objective of the qualitative review is to identify and summarise the evidence on patient experiences of meniscal tears. In particular we will focus on patient experiences of treatment (surgical and non-surgical), treatment pathways and their views of the outcomes used in meniscal tear research.

## Methods and analysis

This study protocol was developed in accordance with the PRISMA-P (Preferred Reporting Items for Systematic review and Meta-Analysis Protocols) checklist. In addition to this manuscript the protocol has been registered on the PROSPERO database and can be found at https://www.crd.york.ac.uk/PROSPERO/display_record.php?RecordID=122179

**Eligibility criteria**

Quantitative review

Inclusion criteria

- Study design
    - Full text Randomised controlled trials in patients treated for a meniscal tear (for example, comparing surgery or a non-operative intervention [exercise therapy, pharmacological therapy or observation] versus a comparator group).



For peer review only - http://bmjopen.bmj.com/site/about/guidelines.xhtml
For peer review only

  - Prospective cohort studies adjusted for case-mix reporting outcomes in patients being treated for a meniscal tear.
- Studies reporting at least one established knee-related PROM score for at least three months.
- English language studies only.

Exclusion criteria

- Studies not reporting any of the clinical outcomes selected for this review.
- Studies reporting outcomes in patients with additional major knee ligament injury or fractures around the knee.
- Abstract or conference publications.

Qualitative review

Inclusion criteria

- Qualitative studies reporting the views and experiences of patients with a meniscal tear, implementing all types of qualitative methodology and method.
- English language studies only.

Exclusion criteria

- Abstract or conference publications

**Search Strategy and Quality assessment**

Following consultation with a librarian a search strategy was devised (see supplementary file). The authors aim to search the following databases MEDLINE; Excerpta Medica Database (EMBASE); Allied and Complementary Medicine (AMED); and Cochrane Central Register of Controlled Trials (CENTRAL) using OVID Sp. We will also search Web of science and sociofile for qualitative studies. Search strategies can be seen in the Supplementary files (Appendix 1 and 2). We will also search reference lists of included studies to identify further citations. All citations will be imported into Endnote X9 reference management software[23] (Clarivate Analytics, Philadelphia, USA). After removal of duplicates, titles and abstracts will be screened according to the inclusion criteria. The full texts of studies potentially meeting the inclusion criteria will be screened by two authors (IA and CK). These authors will independently assess each study and any discrepancies will be addressed by discussion with a senior author (NP or AM).

RCTs will be qualitatively assessed using the Cochrane risk of bias tool[24]. Observational studies will be assessed using the Newcastle-Ottawa scale[25]. Two authors (IA and CK) will independently assess



For peer review only - http://bmjopen.bmj.com/site/about/guidelines.xhtml

the quality of each included study with discrepancies being resolved following discussion with a senior author (NP or AM).

Each qualitative study will be critically appraised using the Critical Appraisal Skills Programme (CASP) for qualitative studies[26]. An in-depth quality assessment of each qualitative study will be performed accounting for validity of the results, appropriateness of the results and the applicability of the results. Each study will be graded as adequate, partially adequate or inadequate. Grading will be performed by two review authors (IA and CK) any discrepancies will be discussed with a senior author (AM). If rated as inadequate the study will be excluded from the meta-synthesis.

Two authors (IA and CK) will independently assess the quality of evidence. We will use the GRADE considerations (study limitations, consistence of effect, imprecision, indirectness and publication bias) to assess the quality of the body of evidence[27]. Decisions to upgrade or downgrade body of evidence will be clearly stated.

**Outcomes**

Primary outcome

The primary outcome measure is knee pain or function measured by at least one of the following validated knee questionnaires: (i) Knee injury and osteoarthritis outcome score (KOOS); (ii) Oxford knee score (OKS); (iii) Western Ontario meniscal evaluation tool (WOMET); (iv) Lysholm score and (v) International Knee Documentation Committee score (IKDC); (vi) Western Ontario and McMaster Universities Osteoarthritis Index (WOMAC) score.

**Data extraction**

*Extraction plan for quantitative studies*

For each study, we will collect number of patients (in each arm if RCT or non-randomised study) and the intervention type, defined as meniscectomy, meniscal repair, placebo surgery or non-operative. In addition, mean age, gender, BMI, method of diagnosis of tear, imaging findings (presence of OA), patient reported symptoms, whether patient and public involvement (PPI) was reported and means and standard deviations (SDs) of the PROMs score at each time-point reported will also be collected. We will also extract information on whether any qualitative data were included in the final study report. If a study does not report mean or SD, we will contact the study authors for further



For peer review only - http://bmjopen.bmj.com/site/about/guidelines.xhtml

information. If the SD is not provided then where possible estimates will be calculated from other reported statistics (data summaries), e.g. a test statistic or P value, using standard methods as described in the Cochrane handbook[24]. If data are not represented in a simple numeric format (e.g. in a table or in the study text), but are presented graphically only (e.g. a figure or graph), two authors (IA and CK) will extract the data manually. If failing this we are unable to extract the data we will contact the study authors and request the data.

*Data analysis*

Outcome scores will be plotted against time from study intervention (e.g. non-operative [exercise therapy or pharmacological therapy] or surgery) to describe change from baseline (at the point of recruitment) to all follow up time points reported in the treatment arms included. This will provide a simple visual representation of the clinical course of the condition. If studies report an observation or a 'watch and wait' arm where no treatment is initiated we will plot this separately. This will provide an insight into the natural history of the condition.

To determine variation in size of response, the change of outcome will be assessed by calculating the bias-corrected standardised mean change (SMC) at 3, 6, 12, 24, 36, 48 and 60 months. The SMC is calculated by subtracting the follow-up mean score for the PROM of interest from the baseline mean score. This is then divided by the pooled SD and multiplied by the bias correction factor based on group size. Follow up or baseline SD will be used if the pooled SD is not reported or provided when study authors are contacted for further information. The 95% confidence intervals (CI) will be calculated using estimates of the variance of the SMC[28].

Studies using a variety of outcomes scales or PROMs can be pooled as the SMC standardises the measurement of change. As in previously reported meta-analyses we will calculate a combined pooled SMC for each time point using a random effects model[20,21]. We will do this for the meniscectomy arm, meniscal repair arms and non-operative arms of studies. A simple correlation analysis (using Pearson's correlation coefficient) on all SMCs between each pair of time points will be used to assess the relationship between intervals.

Should the data allow, we will perform a subgroup analysis based on presence or absence of OA and also based on mechanism of injury. Tears with a clearly defined history of trauma will be compared to those without a clear history of trauma, and tears associated with features of OA will be compared to tears without associated OA. This will allow exploration of change in outcome scores over time in the presence of absence of OA and trauma. Further subgroup analysis will be performed where we will pool PROMs for exercise therapy and pharmacological interventions separately. These



For peer review only - http://bmjopen.bmj.com/site/about/guidelines.xhtml

will describe the clinical course of non-pharmacological and pharmacological interventions, where such data is available.

We will also conduct a separate analysis comparing outcomes for operative interventions versus non operative interventions. A random effects model will be used to pool the standardised mean differences (SMDs) of comparable groups of studies in a meta-analysis. Examples of comparisons which will be made include 1) APM versus non-operative interventions; 2) Meniscal repair versus non-operative interventions and 3) Meniscal repair versus APM.

All analysis will implemented in R[29] (https://www.r-project .org/) using the metafor package[30].

*Extraction plan for qualitative studies*

A prospective data extraction sheet will be designed to collect the following data: author, year of publication, journal, and aim of study, number of participants, data collection methods, theoretical or methodological underpinnings and results.

*Analysis and Meta-synthesis*

If sufficient studies are identified, a meta-synthesis will be performed to translate existing evidence into new theory. Meta-synthesis is a process which involves synthesis of findings from qualitative studies, interpreting the results and generating new intepretations[31]. Each included study will be read and reread to produce a table of first order constructs (direct quotes from the participants in each individual study) and second order constructs (author interpretation of the meaning of individual participant quotes). We will collect data (or quotes) on participant views on treatment options, treatment pathways and the current outcomes used in meniscal tear research. Thematic coding will be used to identify cluster of themes in order to develop third order constructs (Our interpretation of the cluster themes).

**Discussion**

Previous reviews have focussed on comparing the effectiveness of surgical intervention (arthroscopic meniscectomy) versus a placebo or non-operative intervention[12,17]. There has been no review reporting the clinical course or natural history of the condition. This review will provide a



For peer review only - http://bmjopen.bmj.com/site/about/guidelines.xhtml

comprehensive summary of the patient reported outcomes of all patients being managed with a meniscal tear in the literature. Our analysis will provide an insight into the natural history of the condition and the patterns of change in PROMs following operative management. Patient and public involvement in each of the included studies will be also be explored.

There has been no qualitative synthesis in the literature of patient experiences of meniscal tears. This review will explore and summarise the available qualitative evidences on patient experiences of living with a meniscal tear. This will identify if there is an evidence gap which will require the need for further qualitative research in this patient population.

This review will inform future research through developing the understanding of the natural history of patients with a meniscal tear. It will also inform researchers and healthcare providers on patient views and experiences on living with a meniscal tear.

A potential weakness of this review is based on the studies included. Studies may not include an observation arm where there is no treatment initiated. As a result it will be difficult to describe the true natural history of patients with meniscal tears. Another weakness is that we are pooling the results of all patients with meniscal tears where there is the possibility of clinical heterogeneity in the sample. A recent cohort study reported factors such as age, gender and symptom duration did not influence treatment outcome[18]. As a result a decision was taken to group all tears together.

Strengths of the review include its comprehensive nature of its design. We will include patients being managed by both operative and non-operative measures. Studies will be quality assessed using appropriate methodology and the body of evidence will be assessed using the GRADE criteria. A further strength of this piece of work is the incorporation of qualitative data. This will be the first review to summarise the evidence exploring participant views on treatment options and treatment pathways. This will provide further insight for clinicians in order to determine treatment decisions.

**Patient and public involvement**



For peer review only - http://bmjopen.bmj.com/site/about/guidelines.xhtml

A PPI group of 18 patients with knee osteoarthritis or meniscal tears was set up to discuss this study idea. Input was provided on the importance of patient focussed dissemination of the results and the use of lay summaries. A patient member (DS) reviewed aspects of the manuscript, in particular the study abstract and dissemination plans. A reference group will be established with whom key findings of the study will be discussed and interpretations co-produced. A lay summary of the final results of the review will be produced in combination with the PPI reference team and we will make it freely available via a study twitter page or email if required.

**Ethics and dissemination**

Given the study design, no formal ethical approval will be sought to conduct this review. The findings will be disseminated at national and international conferences. The final report will be submitted for peer reviewed publication. A lay summary will be produced and be freely available via the study twitter page.

**Funding**

This protocol represented research funded by the National Institute of Health Research (NIHR) Doctoral Research Fellowship grant awarded to IA, DRF-2018-11-ST2-030. The views expressed in this publication are those of the authors and not necessarily those of the NHS, the National Institute for Health Research or the Department of Health and Social Care.

**Author contributions**

IA: study conception, drafted and reviewed final manuscript

CK: Drafted and reviewed final manuscript

NP: study conception, drafted and reviewed final manuscript

CH: study conception, drafted and reviewed final manuscript

SS: study conception, drafted and reviewed final manuscript

AP: study conception, drafted and reviewed final manuscript

AM: study conception, drafted and reviewed final manuscript

**Competing interests**



For peer review only - http://bmjopen.bmj.com/site/about/guidelines.xhtml

For peer review only

All authors declare no competing interests.

**References**


1. Baker BE, Peckham AC, Pupparo F, Sanborn JC. Review of meniscal injury and associated sports. *Am J Sports Med* 1985; **13**(1): 1-4.
2. Abram SGF, Judge A, Beard DJ, Price AJ. Adverse outcomes after arthroscopic partial meniscectomy: a study of 700 000 procedures in the national Hospital Episode Statistics database for England. *Lancet* 2018; **392**(10160): 2194-202.
3. Hulet C PH, Peretti G, Denti M. Surgery of the Meniscus. 1 ed. Heidelberg New York Dordrecht London: Springer; 2016.
4. Bourne RB, Finlay JB, Papadopoulos P, Andreae P. The effect of medial meniscectomy on strain distribution in the proximal part of the tibia. *J Bone Joint Surg Am* 1984; **66**(9): 1431-7.
5. Freutel M, Seitz AM, Galbusera F, et al. Medial meniscal displacement and strain in three dimensions under compressive loads: MR assessment. *J Magn Reson Imaging* 2014; **40**(5): 1181-8.
6. Abram SGF, Beard DJ, Price AJ. National consensus on the definition, investigation, and classification of meniscal lesions of the knee. *Knee* 2018; **25**(5): 834-40.
7. Poulsen MR, Johnson DL. Meniscal injuries in the young, athletically active patient. *Phys Sportsmed* 2011; **39**(1): 123-30.
8. Rathleff CR, Cavallius C, Jensen HP, et al. Successful conservative treatment of patients with MRI-verified meniscal lesions. *Knee Surg Sports Traumatol Arthrosc* 2015; **23**(1): 178-83.
9. Glyn-Jones S, Palmer AJ, Agricola R, et al. Osteoarthritis. *Lancet* 2015; **386**(9991): 376-87.
10. Uthman OA, van der Windt DA, Jordan JL, et al. Exercise for lower limb osteoarthritis: systematic review incorporating trial sequential analysis and network meta-analysis. *BMJ* 2013; **347**: f5555.
11. Abram SGF, Beard DJ, Price AJ. Arthroscopic meniscal surgery: a national society treatment guideline and consensus statement. *Bone Joint J* 2019; **101-B**(6): 652-9.
12. Thorlund JB, Juhl CB, Roos EM, Lohmander LS. Arthroscopic surgery for degenerative knee: systematic review and meta-analysis of benefits and harms. *Br J Sports Med* 2015; **49**(19): 1229-35.
13. Kise NJ, Risberg MA, Stensrud S, Ranstam J, Engebretsen L, Roos EM. Exercise therapy versus arthroscopic partial meniscectomy for degenerative meniscal tear in middle aged patients: randomised controlled trial with two year follow-up. *BMJ* 2016; **354**: i3740.
14. Sihvonen R, Paavola M, Malmivaara A, et al. Arthroscopic partial meniscectomy versus sham surgery for a degenerative meniscal tear. *N Engl J Med* 2013; **369**(26): 2515-24.
15. Siemieniuk RAC, Harris IA, Agoritsas T, et al. Arthroscopic surgery for degenerative knee arthritis and meniscal tears: a clinical practice guideline. *Br J Sports Med* 2018; **52**(5): 313.
16. Abram SGF, Judge A, Beard DJ, Wilson HA, Price AJ. Temporal trends and regional variation in the rate of arthroscopic knee surgery in England: analysis of over 1.7 million procedures between 1997 and 2017. Has practice changed in response to new evidence? *Br J Sports Med* 2019; **53**(24): 1533-8.
17. Abram SGF, Hopewell S, Monk AP, Bayliss LE, Beard DJ, Price AJ. Arthroscopic partial meniscectomy for meniscal tears of the knee: a systematic review and meta-analysis. *Br J Sports Med* 2019.
18. Pihl K, Ensor J, Peat G, et al. Wild goose chase - no predictable patient subgroups benefit from meniscal surgery: patient-reported outcomes of 641 patients 1 year after surgery. *Br J Sports Med* 2019.
19. Thorlund JB, Englund M, Christensen R, et al. Patient reported outcomes in patients undergoing arthroscopic partial meniscectomy for traumatic or degenerative meniscal tears: comparative prospective cohort study. *BMJ* 2017; **356**: j356.




For peer review only - http://bmjopen.bmj.com/site/about/guidelines.xhtml

20. Khatri C, Ahmed I, Parsons H, et al. The Natural History of Full-Thickness Rotator Cuff Tears in Randomized Controlled Trials: A Systematic Review and Meta-analysis. *Am J Sports Med* 2019; **47**(7): 1734-43.
21. Artus M, van der Windt DA, Jordan KP, Hay EM. Low back pain symptoms show a similar pattern of improvement following a wide range of primary care treatments: a systematic review of randomized clinical trials. *Rheumatology (Oxford)* 2010; **49**(12): 2346-56.
22. Staniszewska S WS. Some of our evidence is missing: conceptualising and using patient evidence in HTA (Chapter 4). . Patient Involvement in Health Technology Assessment Authors of book: Facey K, Ploug Hansen H, Single A Singapore: Springer Nature; 2017.
23. Analytics C. Endnote X9. EndNote X9.2 ed. Philadephia, USA: Clavirate Analytics; 2018.
24. Green S, Higgins J. Cochrane handbook for systematic reviews of interventions. Version; 2005.
25. Stang A. Critical evaluation of the Newcastle-Ottawa scale for the assessment of the quality of nonrandomized studies in meta-analyses. *Eur J Epidemiol* 2010; **25**(9): 603-5.
26. programme CAs. CASP qualitative checklist. programme CAs. CASP qualitative checklist. https://casp-uk.net/wp-content/uploads/2018/01/CASP-Qualitative-Checklist-2018.pdf (accessed 02/09/2019 2019).
27. Guyatt GH, Oxman AD, Vist GE, et al. GRADE: an emerging consensus on rating quality of evidence and strength of recommendations. *BMJ* 2008; **336**(7650): 924-6.
28. Morris SB. Estimating Effect Sizes From Pretest-Posttest-Control Group Designs. *Organizational Research Methods* 2008; **11**(2): 364-86.
29. team RC. R: A language and environment for statistical computing. Vienna, Autria: R Foundation for Statistical Computing; 2018.
30. W V. Conducting meta-analyses in R with the metafor package. *Journal of statistical software* 2010; **36**(3): 1-48.
31. Walsh D, Downe S. Meta-synthesis method for qualitative research: a literature review. *J Adv Nurs* 2005; **50**(2): 204-11.

For peer review only



For peer review only - http://bmjopen.bmj.com/site/about/guidelines.xhtml

**Supplementary file**

**Appendix 1**

Search strategy for quantitative aspect of review

Database: Ovid MEDLINE(R) ALL <1946 to September 17, 2019>

Search Strategy:

--------------------------------------------------------------------------------

1 exp Tibial Meniscus Injuries/ or exp Meniscus/ (8082)

2 (menisc* adj3 (tear* or injur* or damage*)).mp. [mp=title, abstract, original title, name of substance word, subject heading word, floating sub-heading word, keyword heading word, organism supplementary concept word, protocol supplementary concept word, rare disease supplementary concept word, unique identifier, synonyms] (7547)

3 1 or 2 (10876)

4 arthroscopy.mp. or exp Arthroscopy/ (28087)

5 exp Meniscectomy/ or meniscect*.mp. (3054)

6 (menisc* adj3 repair).mp. [mp=title, abstract, original title, name of substance word, subject heading word, floating sub-heading word, keyword heading word, organism supplementary concept word, protocol supplementary concept word, rare disease supplementary concept word, unique identifier, synonyms] (1565)

7 4 or 5 or 6 (30599)

8 (random$ or placebo$ or single blind$ or double blind$ or triple blind$).mp. [mp=title, abstract, original title, name of substance word, subject heading word, floating sub-heading word, keyword heading word, organism supplementary concept word, protocol supplementary concept word, rare disease supplementary concept word, unique identifier, synonyms] (1389493)

9 exp Randomized Controlled Trial/ (490118)

10 Controlled Clinical Trial/ (93257)

11 exp Clinical Trials as Topic/ (330202)

12 Comparative Study/ (1839704)

13 exp Cohort Studies/ (1898633)

14 Observational Study/ (67005)

15 Prospective Studies/ (513619)

16 8 or 9 or 10 or 11 or 12 or 13 or 14 or 15 (4611873)

17 3 and 7 and 16 (2235)

***************************

For peer review only

**Appendix 2**

Search strategy for qualitative aspect of review

Database: Ovid MEDLINE(R) ALL <1946 to September 17, 2019>

Search Strategy:

--------------------------------------------------------------------------------

1 exp Tibial Meniscus Injuries/ or exp Meniscus/ (8082)

2 (menisc* adj3 (tear* or injur* or damage*)).mp. [mp=title, abstract, original title, name of substance word, subject heading word, floating sub-heading word, keyword heading word, organism supplementary concept word, protocol supplementary concept word, rare disease supplementary concept word, unique identifier, synonyms] (7547)

3 1 or 2 (10876)

4 health knowledge, attitudes, practice/ or "patient acceptance of health care"/ or patient satisfaction/ (216398)

5 exp Interview/ (28684)

6 exp "Surveys and Questionnaires"/ (975116)

7 exp Focus Groups/ (27525)

8 4 or 5 or 6 or 7 (1157450)

9 (patient$ adj3 (view$ or opinion$ or awareness or tolerance or perception or persistenc$ or attitude$ or compliance or satisfaction or concern$ or belief$ or feeling$ or position or idea$ or preference$ or choice$)).mp. [mp=title, abstract, original title, name of substance word, subject heading word, floating sub-heading word, keyword heading word, organism supplementary concept word, protocol supplementary concept word, rare disease supplementary concept word, unique identifier, synonyms] (268702)

10 (Discomfort or comfort or inconvenience or bother$4 or trouble or fear$ or anxiety or anxious or worr$3).tw. (334544)

11 8 or 9 or 10 (1567999)

12 3 and 11 (723)

***************************

For peer review only

For peer review only - http://bmjopen.bmj.com/site/about/guidelines.xhtml

**PRISMA-P (Preferred Reporting Items for Systematic review and Meta-Analysis Protocols) 2015 checklist: recommended items to address in a systematic review protocol***

| Section and topic | Item No | Checklist item |
|---|---|---|
| **ADMINISTRATIVE INFORMATION** | | |
| Title: | | |
| Identification | 1a | Identify the report as a protocol of a systematic review – **Found on title page on title**. |
| Update | 1b | If the protocol is for an update of a previous systematic review, identify as such – **Not applicable** |
| Registration | 2 | If registered, provide the name of the registry (such as PROSPERO) and registration number- **Found on Page 2 abstract** |
| Authors: | | |
| Contact | 3a | Provide name, institutional affiliation, e-mail address of all protocol authors; provide physical mailing address of corresponding author – **Found on Page 1 title page** |
| Contributions | 3b | Describe contributions of protocol authors and identify the guarantor of the review – **Found on page 10 author contributions** |
| Amendments | 4 | If the protocol represents an amendment of a previously completed or published protocol, identify as such and list changes; otherwise, state plan for documenting important protocol amendments – **Not applicable** |
| Support: | | |
| Sources | 5a | Indicate sources of financial or other support for the review –**Found on page 10 Funding** |
| Sponsor | 5b | Provide name for the review funder and/or sponsor – **Found on page 10 Funding** |
| Role of sponsor or funder | 5c | Describe roles of funder(s), sponsor(s), and/or institution(s), if any, in developing the protocol – **Found on page 10 Funding** |
| **INTRODUCTION** | | |
| Rationale | 6 | Describe the rationale for the review in the context of what is already known – **Found on page 4 Introduction** |
| Objectives | 7 | Provide an explicit statement of the question(s) the review will address with reference to participants, interventions, comparators, and outcomes (PICO) – **Found on Page 5 objectives** |
| **METHODS** | | |
| Eligibility criteria | 8 | Specify the study characteristics (such as PICO, study design, setting, time frame) and report characteristics (such as years considered, language, publication status) to be used as criteria for eligibility for the review – **Found on page 5 and 6 Eligibility criteria** |
| Information sources | 9 | Describe all intended information sources (such as electronic databases, contact with study authors, trial registers or other grey literature sources) with planned dates of coverage - **Found on page 6 Search strategy and quality assessment** |
| Search strategy | 10 | Present draft of search strategy to be used for at least one electronic database, including planned limits, such that it could be repeated – **Found on page 12/13 Appendix 1/2** |
| Study records: | | |

For peer review only

| | | |
|---|---|---|
| Data management | 11a | Describe the mechanism(s) that will be used to manage records and data throughout the review **– Found on page 6-8 Search strategy and quality assessment** |
| Selection process | 11b | State the process that will be used for selecting studies (such as two independent reviewers) through each phase of the review (that is, screening, eligibility and inclusion in meta-analysis) – **Found on page 6-8** |
| Data collection process | 11c | Describe planned method of extracting data from reports (such as piloting forms, done independently, in duplicate), any processes for obtaining and confirming data from investigators – **Found on page 7-8 Data extraction** |
| Data items | 12 | List and define all variables for which data will be sought (such as PICO items, funding sources), any pre-planned data assumptions and simplifications - **Found on page 7-8 Data extraction** |
| Outcomes and prioritization | 13 | List and define all outcomes for which data will be sought, including prioritization of main and additional outcomes, with rationale **– Found on page 7-8 Outcomes** |
| Risk of bias in individual studies | 14 | Describe anticipated methods for assessing risk of bias of individual studies, including whether this will be done at the outcome or study level, or both; state how this information will be used in data synthesis – **Found on page 6 quality assessment** |
| Data synthesis | 15a | Describe criteria under which study data will be quantitatively synthesised |
| | 15b | If data are appropriate for quantitative synthesis, describe planned summary measures, methods of handling data and methods of combining data from studies, including any planned exploration of consistency (such as $I^2$, Kendall's $\tau$) |
| | 15c | Describe any proposed additional analyses (such as sensitivity or subgroup analyses, meta-regression) |
| | 15d | If quantitative synthesis is not appropriate, describe the type of summary planned<br><br>**All found on page 7-8 Extraction plan** |
| Meta-bias(es) | 16 | Specify any planned assessment of meta-bias(es) (such as publication bias across studies, selective reporting within studies) – **Not applicable** |
| Confidence in cumulative evidence | 17 | Describe how the strength of the body of evidence will be assessed (such as GRADE) – **Found on page 8 Search strategy and quality assessment** |

*** It is strongly recommended that this checklist be read in conjunction with the PRISMA-P Explanation and Elaboration (cite when available) for important clarification on the items. Amendments to a review protocol should be tracked and dated. The copyright for PRISMA-P (including checklist) is held by the PRISMA-P Group and is distributed under a Creative Commons Attribution Licence 4.0.**

*From: Shamseer L, Moher D, Clarke M, Ghersi D, Liberati A, Petticrew M, Shekelle P, Stewart L, PRISMA-P Group. Preferred reporting items for systematic review and meta-analysis protocols (PRISMA-P) 2015: elaboration and explanation. BMJ. 2015 Jan 2;349(jan02 1):g7647.*

For peer review only
